# Association of vision impairment and hearing impairment with encounters in the criminal justice system among children and young people: a systematic review and meta-analysis

**DOI:** 10.1016/j.eclinm.2025.103590

**Published:** 2025-10-23

**Authors:** Mapa Prabhath Piyasena, Kelly Malcolm, Sean Coghlan, Rolvix Patterson, Gianni Virgili, Ving Fai Chan, Peter D. Donnelly, Neil Kennedy, Susan D. Emmett, Thomas Hampton, Eric Umar, Julie-Anne Little, Seena Fazel, Nathan Congdon

**Affiliations:** a Vision and Eye Research Institute, Faculty of Health, Medicine and Social Care, School of Medicine, Anglia Ruskin University, Cambridge, United Kingdom; b Cochlear Centre for Hearing and Public Health, John Hopkins Bloomberg School of Public Health, Baltimore, USA; c School of Medicine, Dentistry and Biomedical Sciences, Queen's University Belfast, Belfast, Northern Ireland, United Kingdom; d Department of Head and Neck Surgery and Communication Sciences, Duke University School of Medicine, North Carolina, USA; e Department NEUROFARBA, University of Florence, Florence, Italy; f Centre for Public Health, Queen's University Belfast, Belfast, Northern Ireland, United Kingdom; g School of Medicine, University of St Andrews, St Andrews, United Kingdom; h Centre for Hearing Health Access, University of Arkansas for Medical Sciences, Little Rock, USA; i Department of Clinical Sciences, Liverpool School of Tropical Medicine, Liverpool, United Kingdom; j Department of Health Systems and Policy, Kamuzu University of Health Sciences, Blantyre, Malawi; k School of Biomedical Sciences, Coleraine Campus, Ulster University, Coleraine, Northern Ireland, United Kingdom; l Department of Psychiatry, University of Oxford, Oxford, United Kingdom; m Department of Preventive Ophthalmology, Zhongshan Ophthalmic Centre, Sun Yat-Sen University, Guangzhou, China; n Orbis International, New York, USA

**Keywords:** Vision impairment, Hearing impairment, Adolescent, Youth, Incarceration, Recidivism

## Abstract

**Background:**

More than 250,000 children <18 years were incarcerated globally in 2020, and 1.5 million cycle through custody annually. We reviewed studies reporting associations of vision/hearing impairment with the criminal justice encounters among young people aged 10–24 years.

**Methods:**

We searched PubMed, EMBASE, PsycINFO, Web of Science, Scopus, Cochrane, legal and social science data bases to identify studies that describe vision impairment/eye disease and hearing impairment/ear disease among incarcerated youth from inception to 1 June 2025. We included studies of any design assessing criminal justice system contacts among young people with such impairments. Study selection, data extraction and evaluation of bias and quality were done by two reviewers. We performed narrative summaries of prevalence due to high heterogeneity and provided a meta-analysis for odds of vision/hearing impairment among incarcerated youth compared to controls. This study is registered with PROSPERO, CRD42022319876.

**Findings:**

We identified 94 eligible full-texts for screening among 10070 assessed. Twenty-three studies (median year of publication 1989) including 34,993 participants (mean age 15.8 years, range 10.2–20.9) were included in analyses. The reported prevalence of vision impairment among incarcerated youth ranged from 1.35% to 66.0% (16 studies), hearing impairment from 1.36% to 50.4% (11 studies). In meta-analysis of four studies providing control samples, odds of hearing impairment were increased among incarcerated youth compared to controls (Odds Ratio [OR] 4.20, 95% CI 1.79–9.86; p < 0.001, I^2^ = 48.4%). Six studies on vision impairment reported a pooled OR of 1.60 (95% CI 0.95–2·70; p = 0.08, I^2^ = 69.9%); leave-one-out meta-analysis found removal of a single outlying study left a highly significant OR (OR 1.90, 95% CI 1.65–2.19; p < 0.001).

**Interpretation:**

The prevalence of vision and hearing impairment are higher among incarcerated youth than the general population, although highly variable. These results highlight the need for screening and treatment of vision and hearing conditions at reception into prisons and follow up on release.

**Funding:**

Co-first author Mapa Prabhath Piyasena was funded by the Wellcome Trust United Kingdom (Grant No: 222490/Z/21/Z) from year May/2022 to April/2024. Co-author Thomas Hampton is supported by the Wellcome Trust United Kingdom (Grant No: 203919/Z/16/Z). Co-author Dr Rolvix Patterson is supported by the NIH NIDCD training grant R25DC020172, NIH Fogarty International Center Grant D43TW009340, and the Duke Hubert Yeargan Center for Global Health.

**Research in context**

**Evidence before this study**

The burden of vision impairment/eye disease and hearing impairment/ear disease among incarcerated children and young people is not well studied, despite the increasing global prevalence of youth imprisonment. This paucity of research limits possible interventions to reduce recidivism through vision and hearing screening and treatment strategies. We searched MEDLINE (Ovid) from inception to 1 November 2021 for systematic reviews and meta-analyses on this topic in our preliminary search and found none. No interventional studies were identified, and only three studies were found in low-and middle-income countries.

**Added value of this study**

This is the first systematic review assessing the association among children and young people between criminal justice system encounters and vision impairment/eye disease and hearing impairment/ear disease (hereafter shortened to vision impairment and hearing impairment). Twenty-three studies were included that enrolled incarcerated persons aged 10–24 years. Overall prevalence of any vision impairment varied widely in incarcerated youth from 1.35% to 66.0% and hearing impairment ranged from 1.36% to 50.4%. We observed substantial heterogeneity (I^2^ >75%) among 23 studies reporting prevalence of vision/hearing impairment and moderate heterogeneity (I^2^ <75%) in nine studies reporting odds ratios (ORs) of vision/hearing impairment compared to controls. Meta analysis of four studies showed significantly increased odds of hearing impairment in incarcerated youth compared to controls (OR 4.20; 95% CI 1.79–9·86). ORs for vision impairment varied from 0.64 to 21.8 (6 studies); leave-one-out meta-analysis revealed that removal of one outlier study left a highly significant pooled OR (OR 1.90, 95% CI 1.65–2.19), with heterogeneity declining to nil.

**Implications of all the available evidence**

The prevalence of vision and hearing impairment are 2–4 times higher among incarcerated youth than the general population, although highly variable. Longitudinal studies are needed to assess the impact of these conditions on recidivism, and randomised controlled trials can help identify effective interventions. Further studies in low-and middle-income countries, where some 70% of the world's incarcerated population resides, are needed.

## Introduction

Children's health and well-being are a priority of the Sustainable Development Goals (SDGs), including the goals of Poverty Reduction (SDG#1), Eliminating Hunger (SDG#2), Good Health (SDG#3) and Quality Education (SDG#4).^1^ The World Health Organization (WHO) recently reported that investments in adolescent health yields a 10-fold return in social and economic benefits and constitute a “*triple dividend*” for adolescents: in the present, during their adult lives and for their future children.^2^

More than 250,000 children aged <18 years globally are held in detention on any given day^3^ and up to 1.5 million cycle through custodial settings each year.^4^ Prolonged imprisonment has significant public health implications.^5^ The mortality rate in previously-detained adolescents is 4.9 times that of the general population,^6^ and detained adolescents suffer higher rates of mental disorders^7^ and both communicable^4^ and non-communicable diseases.^8^ Higher rates of uncorrected refractive errors (34.8% vs 21.9%; p < 0.001)^9^ and higher threshold for pure tone audiometry (binaural median 11 Hz vs 7 Hz; p < 0.001)^10^ have also been reported in adolescent detainees compared to controls. However, the relationship between vision/hearing impairment and risk of incarceration remains unclear.

The WHO reports that 2.2 billion people globally are visually impaired, one billion of whom are affected by preventable or treatable conditions.^11^ Vision impairment disproportionately affects people in low-and middle-income countries (LMICs),^12^ and is the most common disability affecting school-aged children.^13^ Globally, at least 450 million children have an ocular condition requiring treatment, and 90 million live with some degree of sight loss.^14^^,^^15^ The Lancet Commission on Global Eye Health reported that children with vision impairment in LMICs are up to five times less likely to participate in formal education.^16^ Untreated vision impairment in children is associated with reduced school performance^17^ and delays in social and cognitive development, which in turn are common among young people encountering the criminal justice system.^18^ It has been hypothesised that uncorrected vision impairment may contribute to juvenile delinquency.^19^ Prisoners often have restricted access to eye care, particularly in LMICs, further compounding the problem.^20^

The global burden of hearing impairment is substantial, estimated by WHO at 1.6 billion persons.^21^, ^22^, ^23^ Up to 60% of childhood hearing impairment is preventable,^22^ rising to 75% in underserved settings. Childhood hearing loss often leads to poor speech and language development, lower educational attainment, and psychosocial difficulties.^24^ Children with hearing loss are twice as likely to experience behavioural problems as their normal-hearing peers, even after treatment with hearing aids or cochlear implants.^25^ There is a limited understanding of the association between hearing impairment and criminality.

A scoping review has revealed limited evidence concerning the health of incarcerated adolescents.^26^ In the current systematic review, we explored associations between vision and hearing impairment and youth incarceration. This review synthesises and summarises evidence on the prevalence of vision and hearing impairment among incarcerated children and young people aged 10–24 years and calculates odds ratios through a meta-analysis to test the hypothesis that risk of vision and hearing impairment is higher in incarcerated youth than in non-offending comparators. Our results may inform health-based interventions to reduce the socioeconomic burden of youth recidivism.

## Methods

The Cochrane guidance on conducting a systematic review^27^ and the Preferred Reporting Items for Systematic Reviews and Meta-Analysis-Protocols (PRISMA-P 2015)^28^ were used in our protocol preparation. Conduct and reporting followed the Cochrane guidance and main PRISMA statement.^27^, ^28^, ^29^ (Supplementary File 1) The protocol was registered in the International Prospective Register of Systematic Reviews (PROSPERO: CRD42022319876). We carried out a global review of published studies reporting prevalence of vision and hearing impairment among incarcerated young people aged 10–24 years.

### Search strategy and selection criteria

We searched from inception to 1 June 2025 in the databases PubMed, EMBASE (Ovid), PsycINFO (Ovid), Web of Science, Scopus, Cochrane Database of Systematic Reviews (CDSR) and Cochrane Central Register of Controlled Trials (CENTRAL), Academic Search Ultimate (EBSCO), Criminal Justice Abstracts (EBSCO), Sociological Abstracts (ProQUEST) and International Bibliography of the Social Sciences (IBSS). A comprehensive search strategy was developed in consultation with two information specialists to capture a wide range of studies from medical and legal databases. (Supplementary File 2) We searched Google Scholar for grey literature, and reviewed the references cited in eligible studies to identify additional reports.^30^ Study authors were contacted to retrieve data disaggregated by age group.

### Types of studies

We included studies of any design from all countries that: 1) characterised association between vision/hearing impairment and encounters with the criminal justice system; 2) described prevalence of vision and hearing impairment among incarcerated adolescents; or 3) reported rates of access to vision or hearing care. We excluded studies describing only visual or auditory processing disorders. These conditions have different aetiologies and treatments than other causes of vision or hearing impairment.

### Participants

Eligible participants were young people aged 10–24 years with any current or prior encounter with the criminal justice system, including reports of arrest or incarceration. Potential participants were excluded if they were temporarily in remand or committed crimes that did not lead to incarceration.

### Outcome measures

The primary outcomes were: 1) *vision impairment* defined as ≤ 6/12 in the better-seeing eye or presence of any eye disease, untreated or under-treated condition, including refractive errors, strabismus or cataract; and 2) *hearing impairment* according to the WHO definition of hearing threshold >19 dB in the better-hearing ear.^20^ This includes mild hearing loss (>19 dB to < 35 dB), and presence of any ear disease. The definition of hearing loss included the results of both screening and diagnostic testing. Self-reported history of vision or hearing care was a secondary outcome.

### Data collection and analysis

#### Selection of studies

Study screening was conducted using Covidence review management software (Covidence SaaS Enterprise, Melbourne–Australia). Two reviewers (KM, SC) independently screened titles and abstracts retrieved by our searches against the eligibility criteria. Conflicts were resolved through discussion or adjudicated by a third reviewer (PP, RP, GV). Potentially eligible studies were then screened, and conflicts resolved using the same process. Included full-text articles then underwent separate extractions for vision (SC, PP) and hearing (KM, RP) impairment into Microsoft Excel using Cochrane forms and guidelines^31^: including fields for author, year, country, setting, study design, sample size, participant characteristics, type, severity, and definition of vision/hearing impairment, recidivism rates, and self-reported access to vision/hearing health care. The extraction was performed by one reviewer and verified by a second.

#### Assessment of methodological quality

Risk of bias and the quality and characteristics of studies were assessed using an appropriate tool for each study design, as defined by the Joanna Briggs Institute.^32^

### Data synthesis

Study characteristics were first described, including design, country, setting, participant details and type(s) of impairment. We used a descriptive approach to report the prevalence of vision/hearing impairment, due to substantial heterogeneity observed among primary outcomes, with the goal of informing readers on the variability of prevalence figures. We had planned to use meta-analytic techniques to pool prevalence estimates as well as Odds Ratios (ORs) but found high statistical and methodological heterogeneity between prevalence studies and instead reported pooled ORs only for vision and hearing impairment, excluding other ocular or otologic diseases.

### Statistical analysis

Overall and sub-group level heterogeneity was calculated using Cochrane Q and I^2^ statistics. Values of I^2^ exceeding 75% were deemed high for the purposes of this review and precluded aggregating data in a meta-analysis. Statistical heterogeneity was also assessed graphically by examining the variability of individual study estimates as well as overlapping 95% confidence intervals (95% CIs) across studies. Potential sources of heterogeneity were also assessed through subgroup analyses. Odds ratios were used in meta-analyses to assess the associations between vision/hearing impairment and incarceration. The comparison group was non-offending persons of the same sex and age. We fitted random-effect meta-analysis using restricted maximum likelihood (REML) method to estimate the heterogeneity variance in all models i.e., odds ratio summary estimates and leave-one-out analysis. In the leave-one-out meta-analysis approach, studies were removed one at a time to assess whether any study is particularly influential on pooled estimate. Odds ratios were calculated using standard 2x2 data tables and used un-adjusted odds ratios in the meta-analysis, and continuity correction was applied when found zero cells in one study arm, which was an in-built function in the meta-analysis package. Analyses were performed using STATA-SE (Version-18, StataCorp LLC, Texas–USA).

We aimed to explore the publication bias using funnels plots when at least 10 studies are included in the meta-analysis.

### Ethics approval

Ethics approval and informed consent was not required for conducting this systematic review based on review and synthesis of published primary studies.

### Role of the funding source

Co-first author Mapa Prabhath Piyasena was funded by the Wellcome Trust United Kingdom (Grant No: 222490/Z/21/Z) from May 2022 to April 2024. Co-author Thomas Hampton is supported by the Wellcome Trust United Kingdom (Grant No: 203919/Z/16/Z). Wellcome Trust had no role in the study design, data collection, data analysis, data interpretation, or writing of the report. Co-author Dr Rolvix Patterson is supported by the NIH NIDCD training grant R25DC020172, NIH Fogarty International Center Grant D43TW009340, and the Duke Hubert Yeargan Center for Global Health. The content is solely the responsibility of the authors and does not necessarily represent the official views of the National Institutes of Health.

## Results

### Results of searches

**Figure 1:**
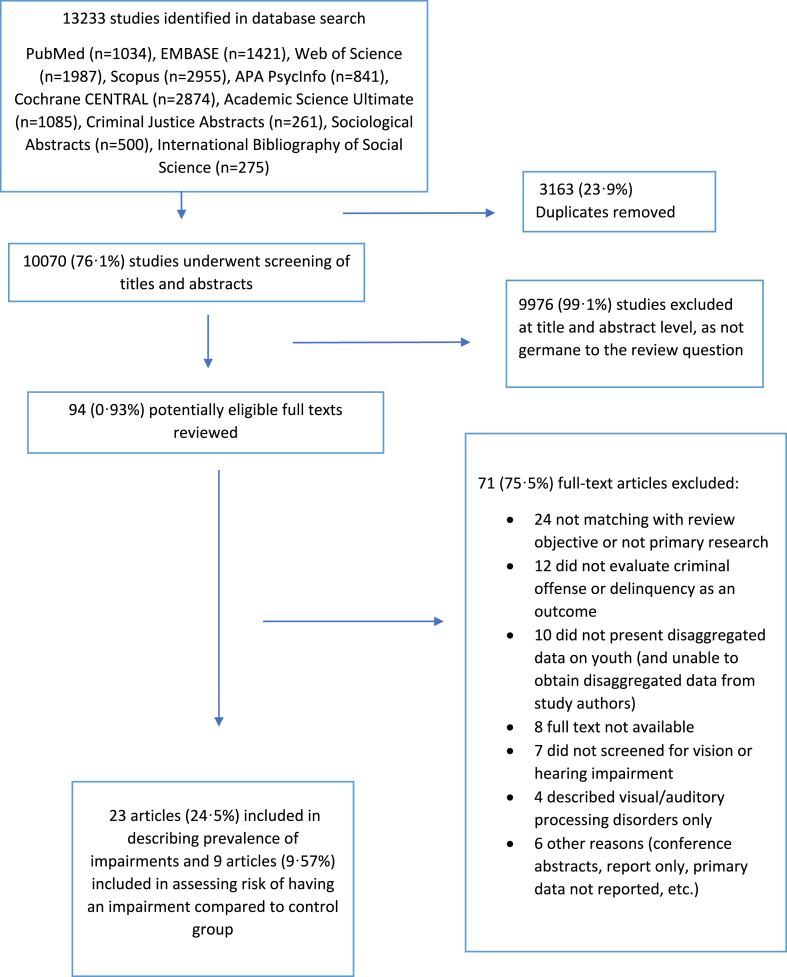
PRISMA Flow Chart: Search strategy and results (according to Preferred Reporting Items for Systematic Review and Meta-analyses).

The database search yielded 13,233 titles and abstracts; 10,070 (76.1%) remained after removing duplicates. Among these, 94 (0.93%) eligible studies were selected for full-text review, of which 71 (75.5%) did not meet inclusion criteria (Fig. 1). The 23 included studies were cross-sectional or observational in design and included one student dissertation^33^ and one national report.^34^ Nine studies compared the disease characteristics of incarcerated adolescents with a matching control sample from the non-offending population.^9^^,^^10^^,^^18^^,^^19^^,^^33^^,^^35^, ^36^, ^37^, ^38^ We retrieved age-stratified data (18–24 years) from one study by contacting the corresponding author.^39^ Incarcerated youth were recruited in all studies as convenience samples from prisons, detention and rehabilitation centres or correctional institutions.

### Characteristics of included studies

Studies were published between 1966 and 2020 (median 1989). The 23 studies included in the synthesis enrolled 34,993 participants (mean sample size n = 2728; range 18–26,740) with a mean age of 14.5 years (range 10.2–20.9 years). The mean proportion of boys was 78.0% (range 49.2%–100%, 21 studies). Fourteen studies included both boys and girls, five studies included only boys,^10^^,^^18^^,^^35^^,^^40^^,^^41^ two only girls^42^^,^^43^ and two^33^^,^^38^ did not report on sex distribution. (Supplementary File 3).

### Study quality and risk of bias assessment

**Figure 2:**
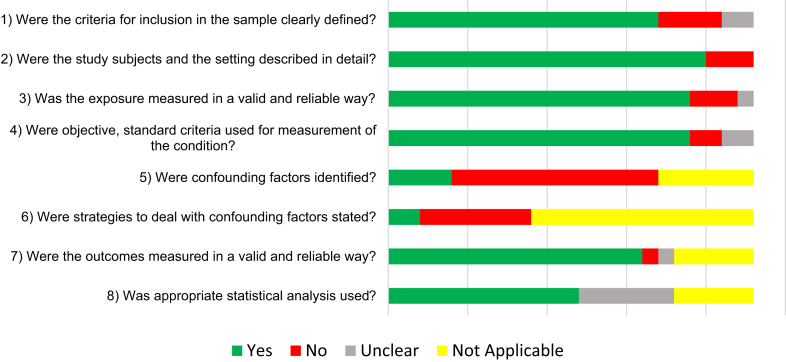
Methodological quality assessment of studies included in the synthesis.

Among the 23 studies assessed for methodological quality and risk of bias (Supplementary File 4), the most common shortcomings were lack of adjustment of confounding factors (56.5%) such as age, sex, level of education and socio-economic status; absence of specified strategies to deal with confounding (30.4%); and insufficient clarity regarding selection criteria for matched controls. Lack of clear inclusion criteria (17.4%), inadequate description of the setting (13.0%), absence of valid and reliable methods to define incarceration (13.0%), and insufficiently clear criteria to measure impairments or outcomes (8.69%) were other domains at risk of bias (Fig. 2).

### Prevalence of vision impairment and eye disease in incarcerated youth

Sixteen studies described prevalence of vision impairment or eye diseases (see Table 1). The sample size of the included sixteen studies varied from 24 to 26,740 with a median of 112. The overall prevalence of any level of vision impairment or eye disease varied widely in incarcerated youth, with a median 22.2% (Interquartile range [IQR] 6.80%–39.0%) and range 1.36% to 66.0%. The largest study, Morgan 1979 (n = 26,740),^37^ reported a prevalence of 1.59%, while the second largest, Harrie 2016 (n = 1632),^9^ found 34.8%. The reasons for high variability in prevalence estimates included variation in visual acuity cut-offs, study designs, definitions of vision impairment, and cadres conducting the vision examination.

**Table 1:** Study characteristics of studies reporting vision impairment or eye disease.

Author, Year, Country	Type of Population and Setting	Sample Size	Mean Age and Age Range	Proportion of Male (%)	Types of Vision Testing		Definition of Vision Impairment (Cut-off Values)	Prevalence of Vision Loss		Prevalence of Other Eye Diseases
					Distance VI	Near VI				
								Refractive Errors/Near Vision Impairment	Distance Vision Impairment	
**1.Barnes 1978 [Ireland]**	Delinquent boys in housing estates [Control sample - Sampling details not reported]	n = 100 [Control n = 115]	Mean N/R [Range: 10–16 years]	100%	Snellen VA^a^	Snellen VA	6/12	Near VI^b^: Worse than 6/12 in 2.0% delinquents and 0% controls	Distance VI: Worse than 6/12 in 3.0% delinquents and 1.0% control	Enucleated eye: 2.0% delinquents and 0% controls
**2.Brian 2011 [Papua New Guinea]**	Prison population [No control sample]	n = 148 (n = 49 young people)	N/R^c^ [Range 18–24 years]	95.9%	Snellen	Near Reading Charts	6/12 (Better eye worse than 6/12)	Near VI (Binocular, N8): 8·1%	Distance VI: 6.1%	N/R (for those <24 years)
**3.Dzik 1966 [USA]**	Juveniles in a detention home under the juvenile court [No control sample]	n = 125	Mean N/R [Range16 years and under]	N/R	N/R	Near Vision at 14″	N/R	Failure of near test: 48.8% (Both Far and Near test failure 35.0%)	Failure of distance test: 50·4%	Overall Failed vision test 72·0%
**4.Harrie 2016 [USA]**	Incarcerated adolescents in a detention centre [Control sample – Middle school students in a public school system ]	n = 1632 [Control n = 1792]	Mean N/R [Range 12–18 years]	69.8%	Snellen VA at 20 feet	N/R	20/40 [Refractive errors: Hyperopia +1.25D^d^ or more, Myopia −1.25D or more· Astigmatism or anisometropia >1.5D]	Uncorrected refractive error: Delinquents 34.8% compared to control public school students 21.9%· [Anisometropia >1.5D n = 29, Astigmatism >1.5D n = 31, Hyperopia > +1.25D n = 9, Myopia > −1·25 D n = 500 (>−4·00D n = 44)]	N/R	Amblyopia 1.35%
**5.Harries 1989 [USA]**	Youth who committed serious crime in a juvenile detention facility [No control sample]	n = 325 to 400 and n = 132 completed testing	Mean N/R [Range 13–19 years]	90.0%	Snellen VA	N/R	20/40 [Convergence near-point test - Reach grasp release testing]	Near VI: 3.78% [Right n = 2, left n = 2, both n = 1] [Average power: Right eye sphere +0.57, Left +0.64, Cylinder −0.27D]	Vision poor than 20/40 (Distance): Average 17.4% [Adjusted prevalence at person level 6.45%] [Right eye n = 8, Left n = 8, Both n = 7]	N/R Strabismus n = 1
**6.Johnson n Zaba 1999 [USA]**	Adjudicated adolescents in a vision screening programme [Control sample – Graduate students]	n = 50 [Control n = 54]	Median age: Adjudicated 19 years and Control 27 years	98.0%	Vision screening battery	N/R	20/40 [Hyperopia:>+1.50D]	VI Near: Offenders 20.0% vs control 0% [Hyperopia Offenders: n = 4 vs control n = 0]	VI Distance: Offenders 4.0% vs control 13.0%	Failed at least one vision test: Offenders n = 37 (74% of participants) vs control n = 32 (59%)
**7.Kaseno 1985 [USA]**	Juvenile delinquents at a rehabilitation centre [No control sample]	n = 1000	Mean: 16.2 years [Range N/R]	90.0%	N/R	N/R	N/R	Refractive errors: 20.0% (n = 451 were given glasses for vision therapy and all with RE never used glasses before)	N/R	N/R
**8.Morgan 1979 [USA]**	Juvenile delinquents in a correctional institution [Control sample – Compared to national level data]	n = 26,740 [Control n = 26,740]	N/R but specified as Children	86.0%	N/R	N/R	N/R	N/R	Visual handicap (VH) n = 422 (1.59% vs control 0.10%) Blind delinquents 0% vs control 0.06%	Any impairment 42.4% (vs National average 12.3%)
**9.Indig 2011 [Australia]** **(NSW Department of Juvenile Justice 2009 Report)**	Young people (rural and urban) in custody [No control sample]	n = 361	Mean 17.0 years [Range: 13–21 years]	88.4%	Snellen VA	N/R	6/12	N/R	Poor eyesight 6.65% (Line 5 correct [6/12] n = 274, 75.9%)	N/R
**10.Ohikhuare 2020 [Nigeria]**	Juvenile delinquents in a juvenile detention centre [Control sample - non-incarcerated children in Lagos aged 9–18 from previously gathered data]	n = 76 (with a n = 76 control sample) [Control n = 76]	Mean age: Male 15.0 years and Female 14.0 years [Range 9–18 years]	N/R	Snellen VA	N/R	Less than 6/6 [Myopia: −0.25D Hyperopia: +0.25 D Astigmatism: Cylindrical Error >0.25 D Anisometropia: 0.50 D]	Refractive errors: Incarcerated 34.2% vs non-incarcerated 44.7% (Astigmatism 32.26% [8·06% incarcerated vs 24.2% control]; Myopia 29% (14.5% incarcerated vs 14.5% control]; Hypermetropia 27.4% [16·1% incarcerated vs 11.3% control])	Vision impairment: less than 6/6: 70% in incarcerated youth	Any ocular morbidity: 2.63% Glaucoma suspect 1.30% Lens opacity 0% Accommodative issues 1.3% Low vision 1.30% Retinal detachment
**11.Oliván Gozalvo 2002 [Spain]**	Delinquent female adolescents in a juvenile correctional facility [No control sample]	n = 35	Mean age: 15.0 years [Range: 14–17 years]	0.0% [100% female]	Optotypes	Optotypes	Requiring referral following visual acuity test with optotypes (including any ocular disorder)	N/R	Vision impaired/ocular disorder present: 5.71% (n = 2)	N/R
**12.Robbins 1983 [USA]**	Adjudicated adolescents referred to a psychiatric clinic by the juvenile judge or probation officer [No control sample]	n = 50 (n = 25, two groups) (in clinic and not in clinic)	Mean age: 15.9 years in clinic sample and 15.7 years in non-clinic sample [Range: 14–18 years]	100.0%	Snellen VA	N/R	20/20	N/R	Vision impaired: 66% less than 20/20 (n = 33) (12% (n = 6) with serious visual pathology [monocular blindness and scotoma]	Inadequate ocular motor performance 18·0% (n = 9) (44% [n = 22] problems with tracking in the left eye, 28% [n = 14] had problems with tracking in right eye, 24% [n = 12] with severe convergence problems)
**13.Shandra 2012 [USA]**	Youth at household level (Youth with a history of arrest included in the review)	n = 7232 [Overall proportion arrested 12.9%]	N/R but specified as adolescents around 16 years of age	50.7%	Vision difficulties self-reported by parents	Vision difficulties self-reported by parents	N/R	N/R	Proportion with VI among the arrested youth: 0.64% (Blindness: Total n = 21 x 28.5%; Vision difficulties Total n = 546 x 9.52%)	Overall prevalence of any sensory impairment (VI or HI) 11%·
**14.Snow 1983 [USA]**	Juvenile Delinquents at a juvenile court centre [Control sample – Compared to a population-based survey done in another study]	n = 253 [Control n = 253]	N/R but specified as juveniles	77.8%	Snellen VA	N/R	20/40 VA 20/40 or poorer acuity with either eye or a two-line difference between the two eyes	Refractive error problems: 24.5% (Male n = 37, Female n = 25)	Overall visual impairment by 20/30: 32.5% (By Eyes: Male n = 98, Female n = 65)	Any ocular problem: 58.0% (Male n = 106, Female n = 40) Ocular muscles imbalances: 41.9% (Male n = 81, Female n = 25)
**15.Weindling 1986 [UK]**	Delinquent adolescents in an intermediate treatment centre [Control sample – non-delinquent children matched to same school, year and postal district]	n = 24 [Control n = 24]	Mean age: Delinquents 15.3 years and control 15.7 years	100.0%	N/R	N/R	N/R	Spectacle wearing: Delinquents n = 1 (lost spectacles) vs Control n = 5	N/R	Failed vision test: Delinquent n = 9 (37.5%) vs Control n = 7 (29%)
**16·Wong 1976 [USA]**	Juvenile delinquents in a juvenile detention centre [Control sample - Compared to a population-based survey done in another study]	n = 633 [Control n = 633]	Mean 14.65 years [Range 7–18 years]	76·9%	N/R	N/R	20/40 or less in either eye [Hyperopia +1.5D or more, Myopia −0.50D or more, Astigmatism ± 1.00D or more, Anisometropia ± 1.00D or more,	Uncorrected Refractive Errors: 21.6% (uRE Distribution: Myopia n = 70 [25.5%]; Myopia with astigmatism n = 96 [35.0%]; Hyperopia n = 16 [5.6%]; Hyperopia with astigmatism n = 72 [26.3%]; Astigmatism n = 18 [6.6%]; Emmetropia n = 2 [0.7%])	Rate of referral based on overall visual impairment 29.4% (n = 186, n = 62 females and n = 124 males)	Other ocular problems: n = 28, 14.1% Ocular muscle coordination issues n = 13, 6.5% Amblyopia n = 7, 3.5%·

a VA-Visual Acuity.

b VI-Vision Impairment.

c N/R-Not Reported.

d D-Dioptre.

Five studies reported distance visual loss, with a range of 3.0% to 50.4%.^35^^,^^36^^,^^39^^,^^40^^,^^44^ Studies mostly used Snellen charts to assess vision, but cut-offs for visual acuity were not uniform. The most common cutoffs were Snellen 6/9, 6/9.5 and 6/12 and LogMAR 0.2 (Snellen 6/9) and 0.3 (Snellen 6/12). Seven studies reported prevalence of near vision impairment or refractive errors, with a range of 2.0% to 34.9%,^9^^,^^19^^,^^33^^,^^35^^,^^36^^,^^39^^,^^45^ but most studies did not report the causes of impairment, since most did not conduct comprehensive eye examinations. The most common reported cause was refractive error. Other causes reported in ten studies (range of 1.59% to 66.0%) including impaired accommodation and ocular muscle disorders.^9^^,^^18^^,^^33^^,^^34^^,^^37^^,^^39^^,^^41^^,^^43^^,^^46^^,^^47^

### Prevalence of hearing impairment and ear disease

Eleven studies described prevalence of hearing impairment or ear disease (Table 2). The sample size of the included eleven studies varied from 24 to 26,740 with a median of 252. Overall prevalence of any level of hearing impairment or ear disease varied widely in incarcerated youth with a median of 19.2% (IQR 7.52%–26.1%) and range of 1.36% to 50.4%. The largest study, Morgan 1979 (n = 26,740),^37^ reported a prevalence of 1.36% while the second largest study He 2019 (n = 1533)^48^ reported a prevalence of 48.1%. Variations in study design, hearing assessment cut-offs and definition of hearing impairment led to these disparate estimates. Excluding hearing impairment, the prevalence of ear disease such middle ear pathology ranged from 6.95% to 9.73% in two studies. The high heterogeneity remained once prevalence was broken by type of auditory defect; thus, we decided not to carry out a meta-analysis and provide a descriptive approach instead.

**Table 2:** Study characteristics of studies reporting hearing impairment or ear disease.

Author, Year, Country	Type of Population and Setting	Sample Size	Mean Age and Age Range	Proportion of Male (%)	Modality				Criteria for Hearing Impairment			Prevalence of Hearing Loss	Prevalence of Other Ear Diseases
					Puretone Audiometry	Otoscopy	Tympanometry	Self-Report	Frequencies Screened/Tested	Normal hearing definition	Other Types of Measurements		
**1.Belenchia, 1983 [USA]**	Prison population [No control sample]	n = 136	Mean age: 20.73 [N = 55 in Range: 16–25 years]	70·6%	X				1, 2, 4, & 6 kHz	Response at ≤ 20 dB HL^a^ at all frequencies	N/A^c^	48.5% (n = 136) for the full age range of participants· [n = 55 up to 25 years, n = 36–26 to 40 years and n = 16 more than 40 years)	30.9% (n = 42) had no response at 25 dB HL at any one frequency 23.9% (n = 32) had no response at 30 dB HL at any one frequency
**2.Cozad 1966 [USA]**	Industrial Schools for institutionalized children [No control sample]	n = 300	Mean age: Male 15.0 years, Female 16.0 years [Range: 10–18 years]	70.6%	X				0.25, 0.5, 1, 2, 3, 4, 6, & 8 kHz	Response at 10 dB HL at 1, 2, & 6 kHz and a response at 4 kHz at 20 dB sensation level	N/A	24.3% (n = 73)	Comprehensive audiometry testing (air and bone) of participants failing the screening was completed and showed 72.6% (n = 53) had sensorineural HI 27.4% (n = 20) had mixed HI None had conductive HI
**3.He 2019 [Australia]**	Survey of Aboriginal children in Australia [No control sample]	n = 1533	N/R	49.2%	X				0·5, 1, & 2 kHz	3-frequency PTA^b^ ≤ 15 dB HL	N/A	48.08% (n = 737)	Targeted age group was around 10 years of age 21.4% (n = 324) had unilateral hearing loss 19.63% (n = 301) had mild HI 7.31% (n = 112) had moderate or worse HI
**4.Holmes 1996 [USA]**	Juvenile delinquents in a juvenile detention center [No control sample]	n = 226	Mean age: 15.3 years [Range: 9–18 years]	76.5%	X	X	X		1, 2, 4, & 6 kHz	Response at 25 dB HL at all frequencies	Jerger type B or C tympanogram; Excessive cerumen impeding a clear view of the tympanum	26.11% (n = 59)	9.7% (n = 22) failed otoscopy 7.5% (n = 17) failed tympanometry
**5.Indig 2011 [Australia]** **(NSW Department of Juvenile Justice 2009 Report)**	Juvenile delinquents from 10 juvenile delinquent centers [No control sample]	n = 278	Mean age: 17.0 years [Range: N/R]	88.0%	X	X					44.24% (n = 123) had abnormal ear canals and 14.03% (n = 39) had abnormal eardrums during otoscopy in at least one ear·	18.35% (n = 51)	32% (n = 89) had unilateral hearing loss Of those with hearing loss (n = 51) 88.24% (n = 45) had mild hearing loss and 11.76% had moderate· No participants were classified as severe
**6.Lount 2017 [New Zealand]**	Juvenile delinquents in Juvenile detention center [Control sample – Randomly selected age, sex matched students from Schools]	n = 33 [Control n = 39]	Mean age: 16.04 years [Range: 14–17 years]	100%	X	X	X	X	0.25, 0.5, 1, 2, 4, & 8 kHz	4 – frequency PTA (0.5, 1, 2, & 4 kHz) ≤15 dB HL	Type B or C tympanogram in either ear· Acoustic ipsilateral reflex threshold at 1 kHz of >100 dB	30.3% (n = 10)	6% (n = 2) of youth offenders vs· no controls had mild hearing loss based on their PTA· For tympanometry, 48% (n = 16) of youth offenders vs· 62% (n = 24) of controls had an abnormal tympanogram in at least one ear For self-report of hearing, 36% (n = 12) of youth offenders vs· 44% (n = 17) of the control group reported hearing as “sometimes difficult” 3% (n = 1) of youth offenders reported hearing as “always difficult”
**7.Morgan 1979 [USA]**	Juvenile delinquents from many juvenile detention centers [Control sample – Compared to national level population-based data]	n = 26,740 [Control n = 26,740]	N/R	86.0%				X	N/A	N/A	N/A	1.36% (n = 365)	N/A
**8.Omokanye 2022 [Nigeria]**	Young adults in a juvenile correctional facility [Control sample – Age, sex matched students from a secondary government school]	n = 135 [Control n = 135]	Mean age: 19.0 years [Range 15–21 years]	N/R	X	X	X	X	4fPTA: Average of threshold at 0.5, 1, 2 and 4 kHz	Cut off for normal hearing threshold ≤25 dB	Conductive hearing loss assessment and self-reported otologic complaints	Prevalence of hearing loss (PTA, air conduction): 19.2% (n = 26) in better and 34.8% (n = 48) in worse ear inmates vs 0·0% (n = 0) in better and 2.2% (n = 13) in worse ear of controls Conductive hearing loss 24.4% (n = 33) inmates vs 9.4% (n = 13) controls Sensory-neural hearing loss 2.2% (n = 3) and mixed hearing loss 9.0% (n = 12) among inmates and 0% in controls	Self-reported hard of hearing: 31.9% (n = 43) inmates vs 8.7% (n = 12) controls Otoscopic Findings: Impacted wax (cerumen) 13.3% (n = 18) inmates vs 7.4% (n = 10) control; perforated or neo-membrane 8.0% (n = 11) inmates vs 0.7% (n = 1) controls; Dull tympanic membrane light reflex 26.0% (n = 35) inmates vs 14.0% (n = 19) controls; Retracted position of tympanic membrane 12.6% (n = 17) inmates vs 0.7% (n = 1) controls
**9.Shandra 2012 [USA]**	Adolescents in the National Longitudinal Survey of Youth 1997 [No control sample]	n = 933	N/R	N/R				X	N/A	N/A	N/A	16.47% (n = 85)	N/A
**10.Wagner 1983 [USA]**	Prisoners in a Treatment center for women [No control sample]	n = 50 [n = 18 < 24 years]	Mean age: 27.64 years [Range: 18–44 years]	0·0%	X				1, 2, & 4 kHz	Response at 20 dB HL at 1 & 2 kHz and 25 dB HL at 4 kHz	N/A	16.0% (n = 8) [Data stratified by age range 18–20 years and 21–23 years]	N/A
**11.Weindling 1986 [UK]**	Juvenile delinquents in a Juvenile detention center [Control sample - Non-delinquent children matched to same school, year and postal district]	n = 24 [Control n = 24]	Mean age: 15.3 years [Range: N/R]	100%	X				0.5, 1, 2 & 4 kHz	Response on at least two frequencies at 30 dB HL	N/A	33.0% (n = 8)	N/A

X, Type of examination done in the study.

a HI-Hearing Impairment.

b PTA-Pure Tone Audiometry.

c N/A-Not Applicable.

In 11 hearing studies, cut-offs for poor hearing were not uniform. Six studies reported the results of screening for hearing loss using pure-tone audiometry, with cut-offs ranging from 10 dB HL to 30 dB HL across frequencies from 500 Hz to 6000 Hz.^18^^,^^38^^,^^42^^,^^49^, ^50^, ^51^ Two studies reported hearing loss using a three or four frequency pure tone average (PTA) from pure tone audiometry,^10^^,^^42^ one study used self-reported hearing,^47^ and two studies had unclear definitions of hearing loss.^34^^,^^37^ In two studies, middle ear status was assessed using tympanometry and middle ear dysfunction was defined as any tympanogram classification other than type A.^10^^,^^51^

### Self-reported history of healthcare access and rate of recidivism

There were no identified studies reporting healthcare service access data for incarcerated youth with vision or hearing impairment, or rates of recidivism among such persons.

### Association of vision and hearing impairment with incarceration

**Figure 3:**
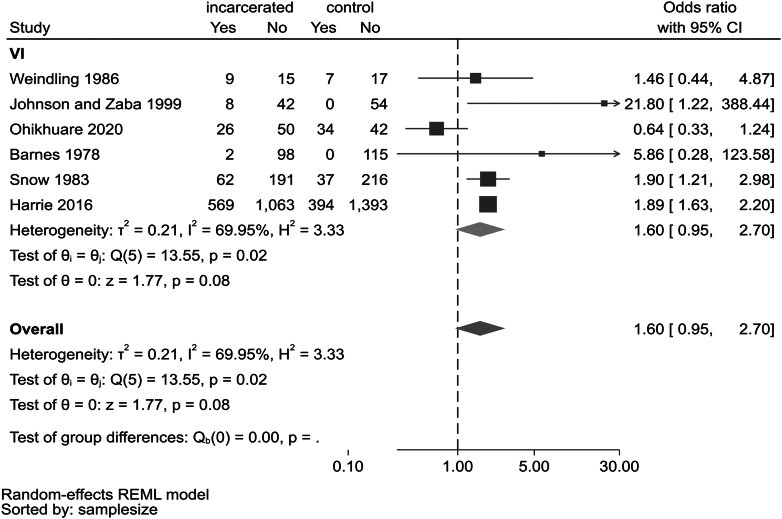
Forest plot of odds of prevalence of vision impairment among the incarcerated youth compared to a non-offender control sample from population.

Nine studies with a control sample (non-offending, age- and sex-matched samples from the population, including schools) were eligible for meta-analysis.^9^^,^^10^^,^^18^^,^^19^^,^^33^^,^^35^, ^36^, ^37^, ^38^ One study reported outcomes for both vision and hearing impairment.^18^ (Supplementary File 5) Meta-analysis of six studies with size 24 to 1632 participants reporting on vision impairment found a pooled OR of 1.60 (95% CI 0·95–2.70; p = 0.08) with high heterogeneity (I^2^ = 69.9%).^9^^,^^18^^,^^19^^,^^33^^,^^35^^,^^36^ (Fig. 3) Leave-one-out meta-analysis found that removal of Ohikhuare 2020^33^ left a highly significant OR (1.90, 95% CI 1.65–2.19, p < 0·0001 with I^2^ = 0). (Supplementary File 6) Potential reasons for discordant effects on refractive errors in this study (i.e., Ohikhuare 2020)^33^ was that astigmatism was more common in non-incarcerated (24.2%), vs incarcerated (8.06%) children; despite the fact that ocular pathologies were more numerous on the latter, and this study became an outlier due to having considered astigmatism separate from refractive error analysis, which other studies did not.

**Figure 4:**
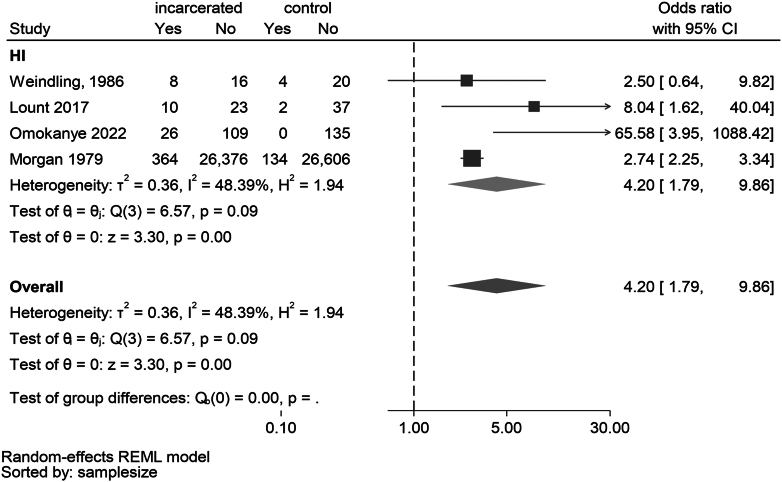
Forest plot of odds of prevalence of hearing impairment among the incarcerated youth compared to a non-offender control sample from population.

Hearing impairment was consistently more prevalent in incarcerated populations in four studies.^10^^,^^18^^,^^37^^,^^38^ The meta-analysis of four studies reporting on hearing impairment found a pooled OR of 4.20 (95% CI 1.79–9.86, p < 0.001) with moderate heterogeneity (I^2^ = 48.4%) (Fig. 4).

Evidence from Morgan 1979^37^ suggested that blindness (OR = 0.03, 95% 0.0–0.50) and deafness (OR = 0.42, 95% CI 0.18–0.96) were both less common among incarcerated youth.

We did not explore the publication bias using funnels plots since less than 10 studies were included in the meta-analysis.

## Discussion

We found evidence suggestive of increased burden of vision and hearing impairment among incarcerated children and young people aged 10–24 years. Specifically, we found an at least two-fold increased odds of vision or hearing impairment among incarcerated youth compared to the similar-aged general population. Although studies on vision impairment and eye diseases had substantial variability in measurement approaches, meta-analysis of four studies reporting solely on hearing impairment found four times higher odds among incarcerated youth compared to controls. These findings are consistent with previous research suggesting that health conditions are more prevalent in the incarcerated youth population.^26^ Sensorineural hearing loss and refractive errors, the former treatable with hearing aids and the latter readily corrected with eyeglasses, were the most common impairments in the reviewed studies. Regarding complete blindness and deafness, evidence from a single large study, conducted in 1979 and comparing incarcerated youth with a reference general population, suggested that these conditions were less common in incarcerated youth, though complete blindness and deafness is rare in young populations.

The $5.7 billion spent annually on incarcerated youths in the United States^52^ underscores the importance of our findings. High levels of crime and violence hinder economic growth and consume scarce public resources. Current evidence highlights failures of existing strategies to reduce youth crime.^53^^,^^54^ Custodial sentences are costly, especially maintaining a young person in a secure unit.^55^ Incarceration can have life-long impacts on a youth's economic and social prospects, creating a major burden for society. The Istanbul Declaration (2007) emphasised the importance of prison health as a public health priority.^56^ Incarceration is associated with significant detrimental health outcomes, particularly early in development.^57^ Incarcerated young offenders have a high prevalence of health conditions,^58^ and unmet healthcare needs^59^ and nearly half (46%) of newly detained children have urgent health needs requiring immediate medical attention.^60^ The interventions to promote education and rehabilitation of offenders may be more successful if they were deployed in with health interventions to maximize success, as an example if an offenders have vision impairment due to an uncorrected refractive error they are going to be disadvantaged in accessing the rehabilitation programmes that implicitly require good vision.

We did not identify any studies reporting links with recidivism. Rates of recidivism, as measured by various levels of re-involvement with the justice system, are typically above 50% over one year post-release for youth.^61^^,^^62^ Most programs aimed at reducing recidivism among youth offenders are based on cognitive and behavioral treatments, and have modest success at best.^63^^,^^64^ There is little evidence globally on health interventions to reduce rates of recidivism.^65^^,^^66^ Some studies suggest that healthcare can contribute to preventing juvenile incarceration.^63^^,^^65^^,^^67^ Underreporting of the number of incarcerated youth and limited data on their health have contributed to neglect of this problem. High quality trials are needed for health interventions, such as vision and hearing treatments, to reduce recidivism among young people.

Limitations include the use of varying definitions of hearing and vision loss among studies, leading to high heterogeneity among the included studies.^68^ The WHO defines hearing loss as a hearing threshold of ≥ 20 dB in the better-hearing ear across 500, 1000, 2000, and 4000Hz frequencies.^20^ To improve the comparability of data, future studies should use the current WHO definitions for vision and hearing impairment. Overall, there were insufficient data to statistically explore possible reasons for heterogeneity and we opted not to conduct meta-analyses of prevalence and present the results descriptively.

Another limitation is that nearly 50% of included studies were conducted more than 30 years ago, most of the studies are from Anglophone countries, majority from the US, with a paucity of data from LMICs; thus, their current applicability and generalisability of the findings are unclear. Many of the studies did not report the data points that are essential for current systematic review question. Definitions of the target conditions were different or unclear and controls were chosen from different sources. Poor reporting of study design elements or statistical measures to address confounding were critical issues identified in our appraisal of study quality and reduced the certainty of our conclusions. As an example, vision and hearing impairments may have causal effects on judiciary outcomes, but can also be a marker of syndromic conditions such as visual and auditory processing disorders that affect behaviour in a more complex way than just through sensory impairments. The effects of behavioural or mental health problems in assessing the vision/hearing outcomes were not possible to explore in this review due to paucity of data in primary studies. Screening of children and young adults who are at increased risk of mental health and behavioural problems may be challenging, however due to their incarcerated state, large losses to follow-up and receipt of referral services can likely be avoided. All these issues should be clearly addressed in future research. Furthermore, there were evidence and gaps in data for children and young adults and most of the studies have provided evidence for adult populations. Furthermore, we could not identify studies exploring the impact of vision/hearing impairment on recidivism. There were insufficient data to present outcomes of this review based on the race and ethnicity.

Most of case control studies provided primary outcomes without adjusting for potential confounding factors such as socio-economic status, which may be necessary, for example, when using controls from among school-going children. Finally, we would recommend the adoption of speech testing in future studies to help differentiate between individuals living with hearing impairment and those with neurological or learning disorders including auditory processing disorder and other neurodevelopmental conditions. Evidence gaps identified through this review and implications for research, policy and practices are given under Table 3.

**Table 3:** Summary of gaps in the evidence base and implications for research, policy and practice.

Gaps in the evidence base on vision and hearing impairments among incarcerated children and young people	Implications for research, policy and practice
- There is insufficient reporting of the number of incarcerated youths. A lack of age-disaggregated data hinders the development of strategies to prevent recidivism. - There is a dearth of data on the prevalence and type of vision and hearing impairments among incarcerated young people who are often excluded from estimates of disease burden. - Very few studies have assessed the association between vision and hearing impairment and youth incarceration in LMICs, where 70% of the global burden falls. - No randomised controlled trials have investigated the impact of vision or hearing treatment on recidivism rates. - There is no uniform definition of youth recidivism. - Regular assessment of the vision and hearing status of incarcerated young people is uncommon, so it is unclear whether these impairments increase in prevalence and severity with increased time in prison. - Longitudinal studies, perhaps using administrative data, should focus on causal relationships between vision and hearing impairments and incarceration, and possible bidirectional effects with socio-economic status.	This review suggests the need to: - Conduct regular screening and early referral for eye and ear diseases among incarcerated youth. - Generate more robust evidence through longitudinal studies on links between incarceration and vision and hearing impairment. - Conduct randomised controlled trials to assess the effectiveness of treatment interventions for vision and hearing impairment in reducing recidivism particularly in LMICs. - Better coordination between juvenile justice systems, education providers and the healthcare system. - Strengthen country level legislations on screening for vision/hearing impairment among children and young adults.

In conclusion, in this systematic review on the prevalence and odds of vision impairment and hearing impairment among incarcerated youth, we found evidence of two-fold increase of vision and four-fold rise in hearing impairment among incarcerated young people aged 10–24 years. However, the cross-sectional, observational nature of identified studies, the scarcity of recent data, and lack of LMIC-specific information need to be addressed in further research. To effectively address this health burden, there is a need for randomised trials evaluating interventions to reduce recidivism by correcting vision and hearing loss among incarcerated children and young people, particularly in LIMCs, where 70% of the global burden falls.

## Contributors

NC, PDD, SE, SF and NK conceptualised the project and drafted the initial concept note. KM and SC conducted the preliminary literature search. Afterwards, PP, KM, SC and RP developed the systematic review protocol/methodology under the guidance of NC, PDD, SE, SF, NK and GV. Information specialists RF and SM developed the search strategy and SM ran the electronic database searches. KM and SC conducted the titles and abstract screening, with conflict resolution by PP for vision and RP for hearing. SC and PP extracted the data from vision articles. KM and RP extracted the data from hearing articles. PP and GV led the data synthesis and reviewing manuscript. PP, KM, SC,RP and GV had full access to data in this review and verified all data included in the synthesis. NC, GV, PDD, SE, SF and NK contributed to conducting the review and interpretation of results. PP and KM wrote the first draft of the manuscript as equal first authors, contributed by SC and RP. NC, PDD, GV, NK, SF, SE, VFC, TH, EU and JAL provided revisions and edited the earlier versions of the manuscript. All authors reviewed and approved the final draft of the manuscript for submission and publication.

## Data sharing statement

All data are included in the manuscript and on-line Supplementary Files. The datasets analysed in this review are available through a reasonable request to the corresponding author.

## Declaration of interests

Co-first author Prabhath Piyasena was funded by Wellcome Trust United Kingdom (Grant No: 222490/Z/21/Z) from year May/2022 to April/2024. Thomas Hampton is supported by the Wellcome Trust United Kingdom (Grant No: 203919/Z/16/Z). Wellcome Trust had no role in the study design, data collection, data analysis, data interpretation, or writing of the report. Co-author Dr Rolvix Patterson is supported by the NIH NIDCD training grant R25DC020172, NIH Fogarty International Center Grant D43TW009340, and the Duke Hubert Yeargan Center for Global Health. The content is solely the responsibility of the authors and does not necessarily represent the official views of the National Institutes of Health.

No authors have any other potential or actual competing interests, financial or otherwise.
