# Supplementary material for: Association of vision impairment and hearing impairment with encounters in the criminal justice system among children and young people: a systematic review and meta-analysis
Source: eClinicalMedicine. 2025 Oct 23;89:103590. doi: 10.1016/j.eclinm.2025.103590 (PMC12593635; doi:10.1016/j.eclinm.2025.103590)
Supplement: Multimedia component 1 [file mmc1.docx]

**Online Supplementary Files**

**Supplementary File 1 - Table 1. Preferred Reporting Items for Systematic review and Meta-Analysis [PRISMA] checklist: recommended items to address in a systematic review**

**Supplementary File 2 - Electronic database search strategy**

**Supplementary File 3 - Table 1. Data extraction table of studies describe vision loss or eye disease among the incarcerated youth**

**Supplementary File 3 - Table 2. Data extraction table of studies describe hearing loss or ear disease among the incarcerated youth**

**Supplementary File 4 - Table 1. Study quality appraisal and risk of bias assessment domains* of studies included in the systematic review**

**Supplementary File 5 - Table 1. Summary of vision impairment/eye disease and hearing impairment/ear disease prevalence data extracted for narrative synthesis and meta-analysis**

**Supplementary File 6 - Forest plot of odds of prevalence of vision/hearing impairment among the incarcerated youth compared to a non-offended control sample from population and leave-one-out meta-analysis of vision impairment**

**Supplementary File 1 - Table 1. Preferred Reporting Items for Systematic review and Meta-Analysis [PRISMA] checklist: recommended items to address in a systematic review**

| **Section and Topic** | **Item #** | **Checklist item** | **Location where item is reported** |
| --- | --- | --- | --- |
| **TITLE** | | |  |
| Title | 1 | Identify the report as a systematic review. | 1 |
| **ABSTRACT** | | |  |
| Abstract | 2 | See the PRISMA 2020 for Abstracts checklist. | 4 |
| **INTRODUCTION** | | |  |
| Rationale | 3 | Describe the rationale for the review in the context of existing knowledge. | 7-8 |
| Objectives | 4 | Provide an explicit statement of the objective(s) or question(s) the review addresses. | 8 |
| **METHODS** | | |  |
| Eligibility criteria | 5 | Specify the inclusion and exclusion criteria for the review and how studies were grouped for the syntheses. | 8-9 |
| Information sources | 6 | Specify all databases, registers, websites, organisations, reference lists and other sources searched or consulted to identify studies. Specify the date when each source was last searched or consulted. | 8 |
| Search strategy | 7 | Present the full search strategies for all databases, registers and websites, including any filters and limits used. | 36-43 |
| Selection process | 8 | Specify the methods used to decide whether a study met the inclusion criteria of the review, including how many reviewers screened each record and each report retrieved, whether they worked independently, and if applicable, details of automation tools used in the process. | 9 |
| Data collection process | 9 | Specify the methods used to collect data from reports, including how many reviewers collected data from each report, whether they worked independently, any processes for obtaining or confirming data from study investigators, and if applicable, details of automation tools used in the process. | 9-10 |
| Data items | 10a | List and define all outcomes for which data were sought. Specify whether all results that were compatible with each outcome domain in each study were sought (e.g. for all measures, time points, analyses), and if not, the methods used to decide which results to collect. | 9 |
|  | 10b | List and define all other variables for which data were sought (e.g. participant and intervention characteristics, funding sources). Describe any assumptions made about any missing or unclear information. | 9-10 |
| Study risk of bias assessment | 11 | Specify the methods used to assess risk of bias in the included studies, including details of the tool(s) used, how many reviewers assessed each study and whether they worked independently, and if applicable, details of automation tools used in the process. | 9 |
| Effect measures | 12 | Specify for each outcome the effect measure(s) (e.g. risk ratio, mean difference) used in the synthesis or presentation of results. | 10 |
| Synthesis methods | 13a | Describe the processes used to decide which studies were eligible for each synthesis (e.g. tabulating the study intervention characteristics and comparing against the planned groups for each synthesis (item #5)). | 10 |
|  | 13b | Describe any methods required to prepare the data for presentation or synthesis, such as handling of missing summary statistics, or data conversions. | 10 |
|  | 13c | Describe any methods used to tabulate or visually display results of individual studies and syntheses. | 10 |
|  | 13d | Describe any methods used to synthesize results and provide a rationale for the choice(s). If meta-analysis was performed, describe the model(s), method(s) to identify the presence and extent of statistical heterogeneity, and software package(s) used. | 10 |
|  | 13e | Describe any methods used to explore possible causes of heterogeneity among study results (e.g. subgroup analysis, meta-regression). | 10 |
|  | 13f | Describe any sensitivity analyses conducted to assess robustness of the synthesized results. | 10 |
| Reporting bias assessment | 14 | Describe any methods used to assess risk of bias due to missing results in a synthesis (arising from reporting biases). | 9 |
| Certainty assessment | 15 | Describe any methods used to assess certainty (or confidence) in the body of evidence for an outcome. | N/A |
| **RESULTS** | | |  |
| Study selection | 16a | Describe the results of the search and selection process, from the number of records identified in the search to the number of studies included in the review, ideally using a flow diagram. | 10 |
|  | 16b | Cite studies that might appear to meet the inclusion criteria, but which were excluded, and explain why they were excluded. | 10-11 |
| Study characteristics | 17 | Cite each included study and present its characteristics. | 10-11 |
| Risk of bias in studies | 18 | Present assessments of risk of bias for each included study. | 11 |
| Results of individual studies | 19 | For all outcomes, present, for each study: (a) summary statistics for each group (where appropriate) and (b) an effect estimate and its precision (e.g. confidence/credible interval), ideally using structured tables or plots. | 11-12 |
| Results of syntheses | 20a | For each synthesis, briefly summarise the characteristics and risk of bias among contributing studies. | 11 |
|  | 20b | Present results of all statistical syntheses conducted. If meta-analysis was done, present for each the summary estimate and its precision (e.g. confidence/credible interval) and measures of statistical heterogeneity. If comparing groups, describe the direction of the effect. | 11-13 |
|  | 20c | Present results of all investigations of possible causes of heterogeneity among study results. | 12 |
|  | 20d | Present results of all sensitivity analyses conducted to assess the robustness of the synthesized results. | 12-13 |
| Reporting biases | 21 | Present assessments of risk of bias due to missing results (arising from reporting biases) for each synthesis assessed. | 11 |
| Certainty of evidence | 22 | Present assessments of certainty (or confidence) in the body of evidence for each outcome assessed. | N/A |
| **DISCUSSION** | | |  |
| Discussion | 23a | Provide a general interpretation of the results in the context of other evidence. | 13 |
|  | 23b | Discuss any limitations of the evidence included in the review. | 14 |
|  | 23c | Discuss any limitations of the review processes used. | 12 |
|  | 23d | Discuss implications of the results for practice, policy, and future research. | 14-15 |
| **OTHER INFORMATION** | | |  |
| Registration and protocol | 24a | Provide registration information for the review, including register name and registration number, or state that the review was not registered. | 8 |
|  | 24b | Indicate where the review protocol can be accessed, or state that a protocol was not prepared. | 8 |
|  | 24c | Describe and explain any amendments to information provided at registration or in the protocol. | 8 |
| Support | 25 | Describe sources of financial or non-financial support for the review, and the role of the funders or sponsors in the review. | 10 |
| Competing interests | 26 | Declare any competing interests of review authors. | 16 |
| Availability of data, code and other materials | 27 | Report which of the following are publicly available and where they can be found: template data collection forms; data extracted from included studies; data used for all analyses; analytic code; any other materials used in the review. | 16-17 |

**Supplementary File 2 - Electronic database search strategy**

**PubMed**

(#1 AND #2 AND #3)

adolescent [mesh] OR youth* [tw] OR teen* [tw] OR adolescen* [tw] OR child [mesh] OR child* [tw] OR "school-age*" [tw]

"Correctional Facility" [tw] OR "Correctional Institution" [tw] OR "Correctional Institutions" [tw] OR "Crime" [tw] OR "Criminal" [tw] OR "Criminal activity" [tw] OR "Criminal Behaviors" [tw] OR "Criminal Conduct" [tw] OR "Criminal Intent" [tw] OR "Criminal Intents" [tw] OR "Criminalities" [tw] OR "Criminality" [tw] OR "Detention Center" [tw] OR "Detention Centers" [tw] OR "Gaol" [tw] OR "Gaols" [tw] OR "Homicide" [tw] OR "Illegal Behavior" [tw] OR "Illegal Behaviors" [tw] OR "Illicit Behavior" [tw] OR "Illicit Behaviors" [tw] OR "Incarcerated" [tw] OR "incarceration" [tw] OR "Jail" [tw] OR "Juvenile Delinquency" [tw] OR "Murder" [tw] OR "Offender" [tw] OR "Offenders" [tw] OR "Penal Institution" [tw] OR "Penal Institutions" [tw] OR "Penitentiaries" [tw] OR "Penitentiary" [tw] OR "Prison" [tw] OR "Prisons " [tw] OR "Rape" [tw] OR "Sex Offense" [tw] OR "Sex offenses" [tw] OR "Sexual Abuse" [tw] OR "Sexual Abuses" [tw] OR "Sexual Assault" [tw] OR "Sexual Assaults" [tw] OR "Stealing" [tw] OR "Theft" [tw] OR "Thefts" [tw] OR "Unlawful Behavior" [tw] OR "Unlawful Behaviors" [tw] OR "Violence" [tw] OR "delinquen*" [tw] OR "detain*" [tw] OR "problem behavior*" [tw]

"Vision Disorders"[Mesh] OR "Amblyopia"[Mesh] OR "Color Vision Defects"[Mesh] OR "Diplopia"[Mesh] OR "Hemianopsia"[Mesh] OR "Photophobia"[Mesh] OR "Scotoma"[Mesh] OR "Vision, Low"[Mesh] OR "Achromatopsia" [tw] OR "Achromatopsias" [tw] OR "Amblyopia" [tw] OR "Amblyopias" [tw] OR "Color Blindness" [tw] OR "Color Vision Defect" [tw] OR "Color Vision Deficiencies" [tw] OR "Color Vision Deficiency" [tw] OR "Day Blindness" [tw] OR "Deutan Defect" [tw] OR "Diminished Vision" [tw] OR "Diplopia" [tw] OR "Diplopias" [tw] OR "Double Vision" [tw] OR "Hemeralopia" [tw] OR "Hemeralopias" [tw] OR "Hemianopia" [tw] OR "Hemianopias" [tw] OR "Hemianopsias" [tw] OR "Hemianopsias" [tw] OR "Lazy Eye" [tw] OR "Lazy Eyes" [tw] OR "Light Sensitivities" [tw] OR "Light Sensitivity" [tw] OR "Low Vision" [tw] OR "Macropsia" [tw] OR "Macropsias" [tw] OR "Metamorphopsia" [tw] OR "Metamorphopsias" [tw] OR "Micropsia" [tw] OR "Micropsias" [tw] OR "Monochromatopsia" [tw] OR "Photophobias" [tw] OR "Polyopsia" [tw] OR "Polyopsias" [tw] OR "Protan Defect" [tw] OR "Quadrantanopia" [tw] OR "Quadrantanopias" [tw] OR "Quadrantanopsia" [tw] OR "Quadrantanopsias" [tw] OR "Reduced Vision" [tw] OR "Scotoma" [tw] OR "Scotomas" [tw] OR "Subnormal Vision" [tw] OR "Tritan Defect" [tw] OR "Vision Disabilities" [tw] OR "Vision Disability" [tw] OR "Vision Disorder" [tw] OR "Visual Disorder" [tw] OR "Visual Disorders" [tw] OR "Visual Impairment" [tw] OR "Visual Impairments" [tw] OR vision [tw] OR "Hearing Disorders"[Mesh:NoExp] OR "Hearing Loss"[Mesh:NoExp] OR "Hearing Loss, Bilateral"[Mesh] OR "Hearing Loss, Conductive"[Mesh] OR "Hearing Loss, Functional"[Mesh] OR "Hearing Loss, High-Frequency"[Mesh] OR "Hearing Loss, Mixed Conductive-Sensorineural"[Mesh] OR "Hearing Loss, Sensorineural"[Mesh] OR "Hearing Loss, Unilateral"[Mesh] OR "Acoustic Trauma" [tw] OR "Bilateral Deafness" [tw] OR "Deafness Bilateral" [tw] OR "Deafness Neurosensory" [tw] OR "Deafness Unilateral" [tw] OR "Distorted Hearing" [tw] OR "Dysacusis" [tw] OR "Graefe Usher Syndrome" [tw] OR "Graefe-Usher Syndrome" [tw] OR "Hallgren Syndrome" [tw] OR "Hearing Disorder" [tw] OR "Hearing Impairment" [tw] OR "Hypoacuses" [tw] OR "Hypoacusis" [tw] OR "Neurosensory Deafness" [tw] OR "Hearing Loss" [tw] OR "Paracousis" [tw] OR "Paracusis" [tw] OR "Presbycuses" [tw] OR "Retinitis Pigmentosa And Congenital Deafness" [tw] OR "Sensoryneural Deafness" [tw] OR "Sudden Deafness" [tw] OR "Transitory Deafness" [tw] OR "Unilateral Deafness" [tw] OR "Usher Syndrome" [tw] OR "Ushers Syndrome" [tw] OR "Usher's Syndrome" [tw] OR hearing [tw] OR "sight loss" [tw] OR "ocular disorder*" [tw] OR "eye disease*" [tw] OR "eye disorder*" [tw] OR "refractive error*" [tw] OR cataract* [tw] OR "Eye Diseases, Hereditary" [Mesh] OR "ear disease*" [tw] OR "otologic disease*" [tw] OR "otitis media" [tw] OR "hard of hearing" [tw] OR "auditory perception" [tw] OR "Refractive error*" [tw] OR myopia [tw] OR hyperopia [tw] OR "low vision" [tw] OR blindness [tw] OR glasses [tw] OR cataract* [tw] OR glaucoma* [tw] OR eye [tw] OR ocular [tw] OR conjunctivitis [tw]

**EMBASE**

#1 AND #2 AND #3

'adolescent'/exp OR youth* OR teen* OR adolescen* OR 'child'/exp OR child* OR 'school age*'

'correctional facility'/exp OR 'offender'/exp OR 'crime'/exp OR 'correctional institution':ti,ab,kw OR 'correctional institutions':ti,ab,kw OR crime:ti,ab,kw OR criminal:ti,ab,kw OR 'criminal activity':ti,ab,kw OR 'criminal behaviors':ti,ab,kw OR 'criminal conduct':ti,ab,kw OR 'criminal intent':ti,ab,kw OR 'criminal intents':ti,ab,kw OR criminalities:ti,ab,kw OR criminality:ti,ab,kw OR 'detention center':ti,ab,kw OR 'detention centers':ti,ab,kw OR gaol:ti,ab,kw OR gaols:ti,ab,kw OR homicide:ti,ab,kw OR 'illegal behavior':ti,ab,kw OR 'illegal behaviors':ti,ab,kw OR 'illicit behavior':ti,ab,kw OR 'illicit behaviors':ti,ab,kw OR incarcerated:ti,ab,kw OR incarceration:ti,ab,kw OR jail:ti,ab,kw OR 'juvenile delinquency':ti,ab,kw OR murder:ti,ab,kw OR offender:ti,ab,kw OR offenders:ti,ab,kw OR 'penal institution':ti,ab,kw OR 'penal institutions':ti,ab,kw OR penitentiaries:ti,ab,kw OR penitentiary:ti,ab,kw OR prison:ti,ab,kw OR prisons:ti,ab,kw OR rape:ti,ab,kw OR 'sex offense':ti,ab,kw OR 'sex offenses':ti,ab,kw OR 'sexual abuse':ti,ab,kw OR 'sexual abuses':ti,ab,kw OR 'sexual assault':ti,ab,kw OR 'sexual assaults':ti,ab,kw OR stealing:ti,ab,kw OR theft:ti,ab,kw OR thefts:ti,ab,kw OR 'unlawful behavior':ti,ab,kw OR 'unlawful behaviors':ti,ab,kw OR violence:ti,ab,kw OR delinquen*:ti,ab,kw OR detain*:ti,ab,kw OR 'problem behavior*':ti,ab,kw

'visual disorder'/de OR 'abnormal vision'/exp OR 'amblyopia'/exp OR 'color blindness'/exp OR 'color vision defect'/exp OR 'day blindness'/exp OR 'diplopia'/exp OR 'scotoma'/exp OR 'visual impairment'/exp OR 'hemianopia'/exp OR 'photophobia'/exp OR 'eye disease'/exp OR 'hearing disorder'/exp OR 'hearing impairment'/exp OR achromatopsia:ti,ab,kw OR achromatopsias:ti,ab,kw OR amblyopia:ti,ab,kw OR amblyopias:ti,ab,kw OR 'color blindness':ti,ab,kw OR 'color vision defect':ti,ab,kw OR 'color vision deficiencies':ti,ab,kw OR 'color vision deficiency':ti,ab,kw OR 'day blindness':ti,ab,kw OR 'deutan defect':ti,ab,kw OR 'diminished vision':ti,ab,kw OR diplopia:ti,ab,kw OR diplopias:ti,ab,kw OR 'double vision':ti,ab,kw OR hemeralopia:ti,ab,kw OR hemeralopias:ti,ab,kw OR hemianopia:ti,ab,kw OR hemianopias:ti,ab,kw OR hemianopsias:ti,ab,kw OR 'lazy eye':ti,ab,kw OR 'lazy eyes':ti,ab,kw OR 'light sensitivities':ti,ab,kw OR 'light sensitivity':ti,ab,kw OR macropsia:ti,ab,kw OR macropsias:ti,ab,kw OR metamorphopsia:ti,ab,kw OR metamorphopsias:ti,ab,kw OR micropsia:ti,ab,kw OR micropsias:ti,ab,kw OR monochromatopsia:ti,ab,kw OR photophobias:ti,ab,kw OR polyopsia:ti,ab,kw OR polyopsias:ti,ab,kw OR 'protan defect':ti,ab,kw OR quadrantanopia:ti,ab,kw OR quadrantanopias:ti,ab,kw OR quadrantanopsia:ti,ab,kw OR quadrantanopsias:ti,ab,kw OR 'reduced vision':ti,ab,kw OR scotoma:ti,ab,kw OR scotomas:ti,ab,kw OR 'subnormal vision':ti,ab,kw OR 'tritan defect':ti,ab,kw OR 'vision disabilities':ti,ab,kw OR 'vision disability':ti,ab,kw OR 'vision disorder':ti,ab,kw OR 'visual disorder':ti,ab,kw OR 'visual disorders':ti,ab,kw OR 'visual impairment':ti,ab,kw OR 'visual impairments':ti,ab,kw OR vision:ti,ab,kw OR 'acoustic trauma':ti,ab,kw OR 'bilateral deafness':ti,ab,kw OR 'deafness bilateral':ti,ab,kw OR 'deafness neurosensory':ti,ab,kw OR 'deafness unilateral':ti,ab,kw OR 'distorted hearing':ti,ab,kw OR dysacusis:ti,ab,kw OR 'graefe usher syndrome':ti,ab,kw OR 'graefe-usher syndrome':ti,ab,kw OR 'hallgren syndrome':ti,ab,kw OR 'hearing disorder':ti,ab,kw OR 'hearing impairment':ti,ab,kw OR hypoacuses:ti,ab,kw OR hypoacusis:ti,ab,kw OR 'neurosensory deafness':ti,ab,kw OR 'hearing loss':ti,ab,kw OR paracousis:ti,ab,kw OR paracusis:ti,ab,kw OR presbycuses:ti,ab,kw OR 'retinitis pigmentosa and congenital deafness':ti,ab,kw OR 'sensoryneural deafness':ti,ab,kw OR 'sudden deafness':ti,ab,kw OR 'transitory deafness':ti,ab,kw OR 'unilateral deafness':ti,ab,kw OR 'usher syndrome':ti,ab,kw OR 'ushers syndrome':ti,ab,kw OR hearing:ti,ab,kw OR 'sight loss':ti,ab,kw OR 'ocular disorder*':ti,ab,kw OR 'eye disease*':ti,ab,kw OR 'eye disorder*':ti,ab,kw OR 'ear disease*':ti,ab,kw OR 'otologic disease*':ti,ab,kw OR 'otitis media':ti,ab,kw OR 'hard of hearing':ti,ab,kw OR 'auditory perception':ti,ab,kw OR 'refractive error*':ti,ab,kw OR myopia:ti,ab,kw OR hyperopia:ti,ab,kw OR 'low vision':ti,ab,kw OR blindness:ti,ab,kw OR glasses:ti,ab,kw OR cataract*:ti,ab,kw OR glaucoma*:ti,ab,kw OR eye:ti,ab,kw OR ocular:ti,ab,kw OR conjunctivitis:ti,ab,kw

**Web of Science**

"Vision Disorders" OR Amblyopia OR "Color Vision Defects" OR Diplopia OR Hemianopsia OR Photophobia OR Scotoma OR "Vision, Low" OR Achromatopsia OR Achromatopsias OR Amblyopia OR Amblyopias OR "Color Blindness" OR "Color Vision Defect" OR "Color Vision Deficiencies" OR "Color Vision Deficiency" OR "Day Blindness" OR "Deutan Defect" OR "Diminished Vision" OR Diplopia OR Diplopias OR "Double Vision" OR Hemeralopia OR Hemeralopias OR Hemianopia OR Hemianopias OR Hemianopsias OR Hemianopsias OR "Lazy Eye" OR "Lazy Eyes" OR "Light Sensitivities" OR "Light Sensitivity" OR "Low Vision" OR Macropsia OR Macropsias OR Metamorphopsia OR Metamorphopsias OR Micropsia OR Micropsias OR Monochromatopsia OR Photophobias OR Polyopsia OR Polyopsias OR "Protan Defect" OR Quadrantanopia OR Quadrantanopias OR Quadrantanopsia OR Quadrantanopsias OR "Reduced Vision" OR Scotoma OR Scotomas OR "Subnormal Vision" OR "Tritan Defect" OR "Vision Disabilities" OR "Vision Disability" OR "Vision Disorder" OR "Visual Disorder" OR "Visual Disorders" OR "Visual Impairment" OR "Visual Impairments" OR vision OR "Hearing Disorders" OR "Hearing Loss" OR "Hearing Loss, Bilateral" OR "Hearing Loss, Conductive" OR "Hearing Loss, Functional" OR "Hearing Loss, High-Frequency" OR "Hearing Loss, Mixed Conductive-Sensorineural" OR "Hearing Loss, Sensorineural" OR "Hearing Loss, Unilateral" OR "Acoustic Trauma" OR "Bilateral Deafness" OR "Deafness Bilateral" OR "Deafness Neurosensory" OR "Deafness Unilateral" OR "Distorted Hearing" OR Dysacusis OR "Graefe Usher Syndrome" OR "Graefe-Usher Syndrome" OR "Hallgren Syndrome" OR "Hearing Disorder" OR "Hearing Impairment" OR Hypoacuses OR Hypoacusis OR "Neurosensory Deafness" OR "Hearing Loss" OR Paracousis OR Paracusis OR Presbycuses OR "Retinitis Pigmentosa And Congenital Deafness" OR "Sensoryneural Deafness" OR "Sudden Deafness" OR "Transitory Deafness" OR "Unilateral Deafness" OR "Usher Syndrome" OR "Ushers Syndrome" OR "Usher's Syndrome" OR hearing OR "sight loss" OR "ocular disorder*" OR "eye disease*" OR "eye disorder*" OR "refractive error*" OR cataract* OR "Eye Diseases, Hereditary" OR "ear disease*" OR "otologic disease*" OR "otitis media" OR "hard of hearing" OR "auditory perception" OR "Refractive error*" OR myopia OR hyperopia OR "low vision" OR blindness OR glasses OR cataract* OR glaucoma* OR eye OR ocular OR conjunctivitis (Topic)

and

"Correctional Facility" OR "Correctional Institution" OR "Correctional Institutions" OR Crime OR Criminal OR "Criminal activity" OR "Criminal Behaviors" OR "Criminal Conduct" OR "Criminal Intent" OR "Criminal Intents" OR Criminalities OR Criminality OR "Detention Center" OR "Detention Centers" OR Gaol OR Gaols OR Homicide OR "Illegal Behavior" OR "Illegal Behaviors" OR "Illicit Behavior" OR "Illicit Behaviors" OR Incarcerated OR incarceration OR Jail OR "Juvenile Delinquency" OR Murder OR Offender OR Offenders OR "Penal Institution" OR "Penal Institutions" OR Penitentiaries OR Penitentiary OR Prison OR Prisons OR Rape OR "Sex Offense" OR "Sex offenses" OR "Sexual Abuse" OR "Sexual Abuses" OR "Sexual Assault" OR "Sexual Assaults" OR Stealing OR Theft OR Thefts OR "Unlawful Behavior" OR "Unlawful Behaviors" OR Violence OR delinquen* OR detain* OR "problem behavior*" (Topic)

and

adolescent OR youth* OR teen* OR adolescen* OR child OR child* OR school-age* (Topic)

Web of Science Core Collection

Editions = A&HCI , BKCI-SSH , BKCI-S , CCR-EXPANDED , ESCI , IC , CPCI-SSH , CPCI-S , SCI-EXPANDED , SSCI

**Scopus**

(INDEXTERMS(adolescent) OR TITLE-ABS-KEY(youth*) OR TITLE-ABS-KEY(teen*) OR TITLE-ABS-KEY(adolescen*) OR INDEXTERMS(child) OR TITLE-ABS-KEY(child*) OR TITLE-ABS-KEY(school-age*))

AND

(TITLE-ABS-KEY("Correctional Facility") OR TITLE-ABS-KEY("Correctional Institution") OR TITLE-ABS-KEY("Correctional Institutions") OR TITLE-ABS-KEY(Crime) OR TITLE-ABS-KEY(Criminal) OR TITLE-ABS-KEY("Criminal activity") OR TITLE-ABS-KEY("Criminal Behaviors") OR TITLE-ABS-KEY("Criminal Conduct") OR TITLE-ABS-KEY("Criminal Intent") OR TITLE-ABS-KEY("Criminal Intents") OR TITLE-ABS-KEY(Criminalities) OR TITLE-ABS-KEY(Criminality) OR TITLE-ABS-KEY("Detention Center") OR TITLE-ABS-KEY("Detention Centers") OR TITLE-ABS-KEY(Gaol) OR TITLE-ABS-KEY(Gaols) OR TITLE-ABS-KEY(Homicide) OR TITLE-ABS-KEY("Illegal Behavior") OR TITLE-ABS-KEY("Illegal Behaviors") OR TITLE-ABS-KEY("Illicit Behavior") OR TITLE-ABS-KEY("Illicit Behaviors") OR TITLE-ABS-KEY(Incarcerated) OR TITLE-ABS-KEY(incarceration) OR TITLE-ABS-KEY(Jail) OR TITLE-ABS-KEY("Juvenile Delinquency") OR TITLE-ABS-KEY(Murder) OR TITLE-ABS-KEY(Offender) OR TITLE-ABS-KEY(Offenders) OR TITLE-ABS-KEY("Penal Institution") OR TITLE-ABS-KEY("Penal Institutions") OR TITLE-ABS-KEY(Penitentiaries) OR TITLE-ABS-KEY(Penitentiary) OR TITLE-ABS-KEY(Prison) OR TITLE-ABS-KEY(Prisons) OR TITLE-ABS-KEY(Rape) OR TITLE-ABS-KEY("Sex Offense") OR TITLE-ABS-KEY("Sex offenses") OR TITLE-ABS-KEY("Sexual Abuse") OR TITLE-ABS-KEY("Sexual Abuses") OR TITLE-ABS-KEY("Sexual Assault") OR TITLE-ABS-KEY("Sexual Assaults") OR TITLE-ABS-KEY(Stealing) OR TITLE-ABS-KEY(Theft) OR TITLE-ABS-KEY(Thefts) OR TITLE-ABS-KEY("Unlawful Behavior") OR TITLE-ABS-KEY("Unlawful Behaviors") OR TITLE-ABS-KEY(Violence) OR TITLE-ABS-KEY(delinquen*) OR TITLE-ABS-KEY(detain*) OR TITLE-ABS-KEY("problem behavior*"))

AND

(INDEXTERMS("Vision Disorders") OR INDEXTERMS(Amblyopia) OR INDEXTERMS("Color Vision Defects") OR INDEXTERMS(Diplopia) OR INDEXTERMS(Hemianopsia) OR INDEXTERMS(Photophobia) OR INDEXTERMS(Scotoma) OR INDEXTERMS("Vision, Low") OR TITLE-ABS-KEY(Achromatopsia) OR TITLE-ABS-KEY(Achromatopsias) OR TITLE-ABS-KEY(Amblyopia) OR TITLE-ABS-KEY(Amblyopias) OR TITLE-ABS-KEY("Color Blindness") OR TITLE-ABS-KEY("Color Vision Defect") OR TITLE-ABS-KEY("Color Vision Deficiencies") OR TITLE-ABS-KEY("Color Vision Deficiency") OR TITLE-ABS-KEY("Day Blindness") OR TITLE-ABS-KEY("Deutan Defect") OR TITLE-ABS-KEY("Diminished Vision") OR TITLE-ABS-KEY(Diplopia) OR TITLE-ABS-KEY(Diplopias) OR TITLE-ABS-KEY("Double Vision") OR TITLE-ABS-KEY(Hemeralopia) OR TITLE-ABS-KEY(Hemeralopias) OR TITLE-ABS-KEY(Hemianopia) OR TITLE-ABS-KEY(Hemianopias) OR TITLE-ABS-KEY(Hemianopsias) OR TITLE-ABS-KEY(Hemianopsias) OR TITLE-ABS-KEY("Lazy Eye") OR TITLE-ABS-KEY("Lazy Eyes") OR TITLE-ABS-KEY("Light Sensitivities") OR TITLE-ABS-KEY("Light Sensitivity") OR TITLE-ABS-KEY("Low Vision") OR TITLE-ABS-KEY(Macropsia) OR TITLE-ABS-KEY(Macropsias) OR TITLE-ABS-KEY(Metamorphopsia) OR TITLE-ABS-KEY(Metamorphopsias) OR TITLE-ABS-KEY(Micropsia) OR TITLE-ABS-KEY(Micropsias) OR TITLE-ABS-KEY(Monochromatopsia) OR TITLE-ABS-KEY(Photophobias) OR TITLE-ABS-KEY(Polyopsia) OR TITLE-ABS-KEY(Polyopsias) OR TITLE-ABS-KEY("Protan Defect") OR TITLE-ABS-KEY(Quadrantanopia) OR TITLE-ABS-KEY(Quadrantanopias) OR TITLE-ABS-KEY(Quadrantanopsia) OR TITLE-ABS-KEY(Quadrantanopsias) OR TITLE-ABS-KEY("Reduced Vision") OR TITLE-ABS-KEY(Scotoma) OR TITLE-ABS-KEY(Scotomas) OR TITLE-ABS-KEY("Subnormal Vision") OR TITLE-ABS-KEY("Tritan Defect") OR TITLE-ABS-KEY("Vision Disabilities") OR TITLE-ABS-KEY("Vision Disability") OR TITLE-ABS-KEY("Vision Disorder") OR TITLE-ABS-KEY("Visual Disorder") OR TITLE-ABS-KEY("Visual Disorders") OR TITLE-ABS-KEY("Visual Impairment") OR TITLE-ABS-KEY("Visual Impairments") OR TITLE-ABS-KEY(vision) OR INDEXTERMS("Hearing Disorders") OR INDEXTERMS("Hearing Loss") OR TITLE-ABS-KEY("Acoustic Trauma") OR TITLE-ABS-KEY("Bilateral Deafness") OR TITLE-ABS-KEY("Deafness Bilateral") OR TITLE-ABS-KEY("Deafness Neurosensory") OR TITLE-ABS-KEY("Deafness Unilateral") OR TITLE-ABS-KEY("Distorted Hearing") OR TITLE-ABS-KEY(Dysacusis) OR TITLE-ABS-KEY("Graefe Usher Syndrome") OR TITLE-ABS-KEY("Graefe-Usher Syndrome") OR TITLE-ABS-KEY("Hallgren Syndrome") OR TITLE-ABS-KEY("Hearing Disorder") OR TITLE-ABS-KEY("Hearing Impairment") OR TITLE-ABS-KEY(Hypoacuses) OR TITLE-ABS-KEY(Hypoacusis) OR TITLE-ABS-KEY("Neurosensory Deafness") OR TITLE-ABS-KEY("Hearing Loss") OR TITLE-ABS-KEY(Paracousis) OR TITLE-ABS-KEY(Paracusis) OR TITLE-ABS-KEY(Presbycuses) OR TITLE-ABS-KEY("Retinitis Pigmentosa And Congenital Deafness") OR TITLE-ABS-KEY("Sensoryneural Deafness") OR TITLE-ABS-KEY("Sudden Deafness") OR TITLE-ABS-KEY("Transitory Deafness") OR TITLE-ABS-KEY("Unilateral Deafness") OR TITLE-ABS-KEY("Usher Syndrome") OR TITLE-ABS-KEY("Ushers Syndrome") OR TITLE-ABS-KEY("Usher's Syndrome") OR TITLE-ABS-KEY(hearing) OR TITLE-ABS-KEY("sight loss") OR TITLE-ABS-KEY("ocular disorder*") OR TITLE-ABS-KEY("eye disease*") OR TITLE-ABS-KEY("eye disorder*") OR TITLE-ABS-KEY("refractive error*") OR TITLE-ABS-KEY(cataract*) OR INDEXTERMS("Eye Diseases") OR TITLE-ABS-KEY("ear disease*") OR TITLE-ABS-KEY("otologic disease*") OR TITLE-ABS-KEY("otitis media") OR TITLE-ABS-KEY("hard of hearing") OR TITLE-ABS-KEY("auditory perception") OR TITLE-ABS-KEY("Refractive error*") OR TITLE-ABS-KEY(myopia) OR TITLE-ABS-KEY(hyperopia) OR TITLE-ABS-KEY("low vision") OR TITLE-ABS-KEY(blindness) OR TITLE-ABS-KEY(glasses) OR TITLE-ABS-KEY(cataract*) OR TITLE-ABS-KEY(glaucoma*) OR TITLE-ABS-KEY(eye) OR TITLE-ABS-KEY(ocular) OR TITLE-ABS-KEY(conjunctivitis))

**APAPsycInfo**

S1 AND S2

S1 AND S2

( DE "Hemianopia" OR DE "Vision Disorders" OR DE "Blindsight" OR DE "Partially Sighted" OR DE "Eye Disorders" OR DE "Amblyopia" OR DE "Cataracts" OR DE "Color Blindness" OR DE "Glaucoma" OR DE "Nystagmus" OR DE "Refraction Errors" OR DE "Strabismus" OR DE "Tunnel Vision" OR DE "Hearing Disorders" OR DE "Hearing Loss" OR DE "Hearing Loss" OR DE "Deaf" ) OR TI ( "Vision Disorders" OR Amblyopia OR "Color Vision Defects" OR Diplopia OR Hemianopsia OR Photophobia OR Scotoma OR "Vision, Low" OR Achromatopsia OR Achromatopsias OR Amblyopia OR Amblyopias OR "Color Blindness" OR "Color Vision Defect" OR "Color Vision Deficiencies" OR "Color Vision Deficiency" OR "Day Blindness" OR "Deutan Defect" OR "Diminished Vision" OR Diplopia OR Diplopias OR "Double Vision" OR Hemeralopia OR Hemeralopias OR Hemianopia OR Hemianopias OR Hemianopsias OR Hemianopsias OR "Lazy Eye" OR "Lazy Eyes" OR "Light Sensitivities" OR "Light Sensitivity" OR "Low Vision" OR Macropsia OR Macropsias OR Metamorphopsia OR Metamorphopsias OR Micropsia OR Micropsias OR Monochromatopsia OR Photophobias OR Polyopsia OR Polyopsias OR "Protan Defect" OR Quadrantanopia OR Quadrantanopias OR Quadrantanopsia OR Quadrantanopsias OR "Reduced Vision" OR Scotoma OR Scotomas OR "Subnormal Vision" OR "Tritan Defect" OR "Vision Disabilities" OR "Vision Disability" OR "Vision Disorder" OR "Visual Disorder" OR "Visual Disorders" OR "Visual Impairment" OR "Visual Impairments" OR vision OR "Hearing Disorders" OR "Hearing Loss" OR "Acoustic Trauma" OR "Bilateral Deafness" OR "Deafness Bilateral" OR "Deafness Neurosensory" OR "Deafness Unilateral" OR "Distorted Hearing" OR Dysacusis OR "Graefe Usher Syndrome" OR "Graefe-Usher Syndrome" OR "Hallgren Syndrome" OR "Hearing Disorder" OR "Hearing Impairment" OR Hypoacuses OR Hypoacusis OR "Neurosensory Deafness" OR "Hearing Loss" OR Paracousis OR Paracusis OR Presbycuses OR "Retinitis Pigmentosa And Congenital Deafness" OR "Sensoryneural Deafness" OR "Sudden Deafness" OR "Transitory Deafness" OR "Unilateral Deafness" OR "Usher Syndrome" OR "Ushers Syndrome" OR "Usher's Syndrome" OR hearing OR "sight loss" OR "ocular disorder*" OR "eye disease*" OR "eye disorder*" OR "refractive error*" OR cataract* OR "ear disease*" OR "otologic disease*" OR "otitis media" OR "hard of hearing" OR "auditory perception" OR "Refractive error*" OR myopia OR hyperopia OR "low vision" OR blindness OR glasses OR cataract* OR glaucoma* OR eye OR ocular OR conjunctivitis ) OR AB ( "Vision Disorders" OR Amblyopia OR "Color Vision Defects" OR Diplopia OR Hemianopsia OR Photophobia OR Scotoma OR "Vision, Low" OR Achromatopsia OR Achromatopsias OR Amblyopia OR Amblyopias OR "Color Blindness" OR "Color Vision Defect" OR "Color Vision Deficiencies" OR "Color Vision Deficiency" OR "Day Blindness" OR "Deutan Defect" OR "Diminished Vision" OR Diplopia OR Diplopias OR "Double Vision" OR Hemeralopia OR Hemeralopias OR Hemianopia OR Hemianopias OR Hemianopsias OR Hemianopsias OR "Lazy Eye" OR "Lazy Eyes" OR "Light Sensitivities" OR "Light Sensitivity" OR "Low Vision" OR Macropsia OR Macropsias OR Metamorphopsia OR Metamorphopsias OR Micropsia OR Micropsias OR Monochromatopsia OR Photophobias OR Polyopsia OR Polyopsias OR "Protan Defect" OR Quadrantanopia OR Quadrantanopias OR Quadrantanopsia OR Quadrantanopsias OR "Reduced Vision" OR Scotoma OR Scotomas OR "Subnormal Vision" OR "Tritan Defect" OR "Vision Disabilities" OR "Vision Disability" OR "Vision Disorder" OR "Visual Disorder" OR "Visual Disorders" OR "Visual Impairment" OR "Visual Impairments" OR vision OR "Hearing Disorders" OR "Hearing Loss" OR "Acoustic Trauma" OR "Bilateral Deafness" OR "Deafness Bilateral" OR "Deafness Neurosensory" OR "Deafness Unilateral" OR "Distorted Hearing" OR Dysacusis OR "Graefe Usher Syndrome" OR "Graefe-Usher Syndrome" OR "Hallgren Syndrome" OR "Hearing Disorder" OR "Hearing Impairment" OR Hypoacuses OR Hypoacusis OR "Neurosensory Deafness" OR "Hearing Loss" OR Paracousis OR Paracusis OR Presbycuses OR "Retinitis Pigmentosa And Congenital Deafness" OR "Sensoryneural Deafness" OR "Sudden Deafness" OR "Transitory Deafness" OR "Unilateral Deafness" OR "Usher Syndrome" OR "Ushers Syndrome" OR "Usher's Syndrome" OR hearing OR "sight loss" OR "ocular disorder*" OR "eye disease*" OR "eye disorder*" OR "refractive error*" OR cataract* OR "ear disease*" OR "otologic disease*" OR "otitis media" OR "hard of hearing" OR "auditory perception" OR "Refractive error*" OR myopia OR hyperopia OR "low vision" OR blindness OR glasses OR cataract* OR glaucoma* OR eye OR ocular OR conjunctivitis )

DE "Correctional Institutions" OR DE "Prisons" OR DE "Reformatories" OR DE "Halfway Houses" OR DE "Incarceration" OR DE "Maximum Security Facilities" OR DE "Crime" OR DE "Arson" OR DE "Criminal Record" OR DE "Cybercrime" OR DE "Driving Under the Influence" OR DE "Human Trafficking" OR DE "Illegal Drug Distribution" OR DE "Kidnapping" OR DE "Perpetrators" OR DE "Criminal Offenders" OR DE "Serial Crime" OR DE "Serial Homicide" OR DE "Sex Offenses" OR DE "Incest" OR DE "Rape" OR DE "Sexual Abuse" OR DE "Sexual Coercion" OR DE "Theft" OR DE "Shoplifting" OR DE "Vandalism" OR DE "Violent Crime" OR DE "Criminal Behavior" OR DE "Juvenile Delinquency" OR DE "Criminal Offenders" OR DE "Female Criminal Offenders" OR DE "Male Criminal Offenders" OR DE "Mentally Ill Offenders" OR DE "Gangs" OR DE "Juvenile Gangs" OR DE "Stalking" OR TI("Correctional Facility" OR "Correctional Institution" OR "Correctional Institutions" OR Crime OR Criminal OR "Criminal activity" OR "Criminal Behaviors" OR "Criminal Conduct" OR "Criminal Intent" OR "Criminal Intents" OR Criminalities OR Criminality OR "Detention Center" OR "Detention Centers" OR Gaol OR Gaols OR Homicide OR "Illegal Behavior" OR "Illegal Behaviors" OR "Illicit Behavior" OR "Illicit Behaviors" OR Incarcerated OR incarceration OR Jail OR "Juvenile Delinquency" OR Murder OR Offender OR Offenders OR "Penal Institution" OR "Penal Institutions" OR Penitentiaries OR Penitentiary OR Prison OR Prisons OR Rape OR "Sex Offense" OR "Sex offenses" OR "Sexual Abuse" OR "Sexual Abuses" OR "Sexual Assault" OR "Sexual Assaults" OR Stealing OR Theft OR Thefts OR "Unlawful Behavior" OR "Unlawful Behaviors" OR Violence OR delinquen* OR detain* OR "problem behavior*") OR AB ("Correctional Facility" OR "Correctional Institution" OR "Correctional Institutions" OR Crime OR Criminal OR "Criminal activity" OR "Criminal Behaviors" OR "Criminal Conduct" OR "Criminal Intent" OR "Criminal Intents" OR Criminalities OR Criminality OR "Detention Center" OR "Detention Centers" OR Gaol OR Gaols OR Homicide OR "Illegal Behavior" OR "Illegal Behaviors" OR "Illicit Behavior" OR "Illicit Behaviors" OR Incarcerated OR incarceration OR Jail OR "Juvenile Delinquency" OR Murder OR Offender OR Offenders OR "Penal Institution" OR "Penal Institutions" OR Penitentiaries OR Penitentiary OR Prison OR Prisons OR Rape OR "Sex Offense" OR "Sex offenses" OR "Sexual Abuse" OR "Sexual Abuses" OR "Sexual Assault" OR "Sexual Assaults" OR Stealing OR Theft OR Thefts OR "Unlawful Behavior" OR "Unlawful Behaviors" OR Violence OR delinquen* OR detain* OR "problem behavior*")

**Cochrane Library - CDSR and CENTRAL**

1.Vision Disorders OR Amblyopia OR Color Vision Defects OR Vision Low OR Amblyopia OR Color Blindness OR Color Vision Defect OR Color Vision Deficiencies OR Color Vision Deficiency OR Diminished Vision OR Double Vision OR Lazy Eye OR Lazy Eyes OR Low Vision OR Reduced Vision OR Subnormal Vision OR Vision Disabilities OR Vision Disability OR Vision Disorder OR Visual Disorder OR Visual Disorders OR Visual Impairment OR Visual Impairments OR vision OR Hearing Disorders OR Hearing Loss OR Hearing Loss Bilateral OR Hearing Loss Conductive OR Hearing Loss Functional OR Hearing Loss High Frequency OR Hearing Loss Mixed OR Hearing Loss Unilateral OR Bilateral Deafness OR Deafness Bilateral OR Deafness Unilateral OR Distorted Hearing OR Dysacusis OR Hearing Disorder OR Hearing Impairment OR Hearing Loss OR Unilateral Deafness OR hearing OR sight loss OR ocular disorder OR eye disease OR eye disorder OR refractive error OR cataract OR Eye Diseases Hereditary OR ear disease OR otologic disease OR otitis media OR hard of hearing OR auditory perception OR Refractive error OR myopia OR hyperopia OR low vision OR blindness OR glasses OR cataract OR glaucoma OR eye OR ocular in Title Abstract Keyword

AND

Correctional Facility OR Correctional Institution OR Correctional Institutions OR Crime OR Criminal OR Criminal activity OR Criminal Behaviors OR Criminal Conduct OR Criminal Intent OR Criminal Intents OR Criminalities OR Criminality OR Detention Center OR Detention Centers OR Gaol OR Gaols OR Homicide OR Illegal Behavior OR Illegal Behaviors OR Illicit Behavior OR Illicit Behaviors OR Incarcerated OR incarceration OR Jail OR Juvenile Delinquency OR Murder OR Offender OR Offenders OR Penal Institution OR Penal Institutions OR Penitentiaries OR Penitentiary OR Prison OR Prisons OR Rape OR Sex Offense OR Sex offenses OR Sexual Abuse OR Sexual Abuses OR Sexual Assault OR Sexual Assaults OR Stealing OR Theft OR Thefts OR Unlawful Behavior OR Unlawful Behaviors OR Violence OR delinquent OR detain OR problem behavior in Title Abstract Keyword

AND

3.Adolescent OR youth OR teen OR adolescen* OR child OR child* OR school-age* OR Children OR Young Adults OR Pupils in Title Abstract Keyword - (Word variations have been searched)

**Other electronic databases**

**Academic Search Ultimate**

**Criminal Justice Abstracts**

**Sociological Abstracts**

**International Bibliography of the Social Sciences**

**Supplementary File 3 - Table 1. Data extraction table of studies describe vision loss or eye disease among the incarcerated youth**

| **Author, Year, Country** | **Type of Population and Setting** | **Sample Size** | **Mean Age and Age Range** | **Proportion of Male** | **Definition/s of Vision Impairment** | **Prevalence of Vision Impairment or Ocular Conditions** | **Healthcare Access Data** |
| --- | --- | --- | --- | --- | --- | --- | --- |
| **1.Barnes 1978 [Ireland]** | Delinquent boys in housing estates | n=100 | Mean N/R (10-16 years) | 100·00% | Colour vision - Ishihara's Test Near vision worse than 6/12 Distance vision worse than 6/12 | Colour vision: Control - 09% colour blind, Delinquent only n=1 colour blind Near vision:  Worse than 6/12 in 2% delinquents and 0% controls  Distance vision:  Worse than 6/12 in 3% delinquents and 1% control Enucleated eye 2% delinquents and 0% controls | 13 hospitalised for minor ailments (parents tend to abandon them in hospitals) |
| **2.Brian 2011 [Papua New Ginea]** | Prisoners in Beon Prison | n=148 (n=49 <24 years) | Mean 21 years  (18-24 years) | 95·9% | Presenting distance VA (uncorrected) in better eye worse than 6/12  Presenting near vision (uncorrected) binocular worse than N8 | Presenting distance VA (uncorrected) Right worse than 6/12: n=3 (6·1%), Left worse than 6/12: n=4 (8·1%), better eye worse than 6/12: n=3* (6·1%);  Presenting near vision (uncorrected) binocular worse than N8: n=4 (8·1%)  Colour vision abnormal (if both eyes can see at least N24 and each correctly identified 9 or less of 13 Ishihara plates: 3 (3/48: 6·3%)  [*Age stratified primary data provided by study authors] | N/R |
| **3.Dzik 1966 [USA]** | Juveniles in a detention home under the juvenile court | n=125 | Mean N/R (16 years and under) | N/R | Either failure in test for far seeing or near seeing at 14 inches or perception (getting meaning from what is seeing) | Far vision (n=125 tested) n=63 (54%) failed·  Reading level (n=101 tested) 94% failed·  Comprehension (n=101 tested) 94% failed·  Near vision (n=125 tested) n=61 failed (48%) Both Far and Near (n=125 tested) n=44 failed (35%) Perception (n=121 tested) n=64 failed (53%)  Failed vision test (n=125 tested) n=90 failed (72%) | N/R |
| **4.Harrie 2016 [USA]** | Incarcerated adolescents in a detention centre | n=1632 | Mean N/R (12 - 18 years) | 69·80% | Snellen acuity chart 20 feet, could not see 20/40 as VI  Refractive errors: Hyperopia +1·25 or more, myopia -1·25 or more· Astigmatism or anisometopia >1·5 dioptres | Uncorrected refractive error 34·87% (n=569) compared to control public school students 21·99% (394/1792) Difference in RE - OR 1·9 (95% CI - not given ) (p<0·001) (Incarcerated adolescents 1·9 more likely to require corrective lenses comparable to a sample in a public school system Anisometropia >1·5D n=29 Astigmatism >1·5D n=31 Hyperopia > +1·25D n=9 Myopia > -1·25 D n=500 (> -4·00D n=44) Amblyopia n=22 Another n=492 girls were screened and given glasses  Control - Public schools - n=1792 (21·99%) screened and n=394 referred for eye care· | N/R |
| **5.Harries 1989 [USA]** | Youth who committed serious crime (average 3·42 offences) in a juvenile detention facility | n=325 to 400 and n=132 completed testing | Mean N/R (13 - 19 years) | 90·00% | Vision poor than 20/40 and other functional vision assessments including colour vision | Saccadic test:  n=95 slower than norm (39·4 to 71·9% failed the test) Visual tracing test:  n=95 slower than norm (53 to 71·9% failed the test) Poor than 20/40 (Distant):  Right eye n=8, Left n=8, Both n=7 Poor than 20/40 (Near): Right n=2, left n=2, both n=1 Colour vision abnormal 4/132 Average power right eye sphere +0·57, left +0·64 Cylinder -0·27D | N/R |
| **6.Johnson n Zaba 1999 [USA]** | Adjudicated adolescents in a vision screening programme | n=50 and n=54 control (graduate students) | Median - Adjudicated 19 years and Control 27 years | 98·00% | Vision screening battery administered· VA distance: 20/40 worse as failed vision test VA near: same as for distance Hyperopia: >+1·50D Colour vision: Keystone colour cards | VA Near Offenders n=8 vs control n=0 VA Distance Offenders n=4 vs control n=13 Colour vision Offenders n=8 vs control n=2 Hyperopia Offenders n=4 vs control n=0 Failed at least one vision test Offenders n=37 (74% of participants) vs control n=32 (59%)   (Chi square test showed juvenile offenders scored significantly low visual acuity-near and colour vision sub-sets)· | N/R |
| **7.Kaseno 1985 [USA]** | Juvenile delinquents at a rehabilitation centre | n=1000 | 16·2 years  (Range N/R) | 90·00% | Refractive error, astigmatism or visual stress | Abnormal visual perceptual systems 95%· n=451 were given glasses for vision therapy and 20% were refractive error (All with RE never used glasses before) other 80% therapeutic·  [Rate of Recidivism - Normal rate of re-arrest 50-60%·  Those who received visual therapy re-arrest 10% (in 36 months) (Visual therapy - Training of visualization, imaginary, memory, localization and directionality)] | All with RE have never used glasses before |
| **8.Morgan 1979 [USA]** | Juvenile delinquents in a correctional institution | n=26740 | N/R but specified as Children | 86·00% | (Unclear) | Any impairment 42·4% (National average 12·3%) (Male n=9084, Female n=1431) (n=11,333/26,740) Visually handicap (VH) n=422 (1·59% vs control 0·10%) Blind (B) n=0 vs control 0·06% Learning disabilities 10·59% (VI not specified) | N/R |
| **9.Indig 2011**  **[Australia]**  **(NSW Deparmentt of Juvenile Justice 2009 Report)** | Young people (rural and urban) in custody | n=361 | Mean 17·0 years (13 - 21 years)  [<16 yrs n=129 16·5 to 17·9 yrs n=143 >=18 yrs n=89] | 88·40% | As informed by the examining doctors and visually impaired   Number of lines correctly read, and VA cut off 6/12 | Poor eyesight 7·5% (n=24)   Line 5 correct (6/12) n=274 | Current intake of prescribed medication n=310 (36·8%)  Number not seen a dentist in past year n=140  Childhood vaccination not received (1·1% female and 7·5% male)  Not attended any health service - 12% Having seen a nurse 94%, doctor 77%, psychologist 57%, counsellor 54%  Ever attended a health educational programme - No - male 54·5%, female 42·5% |
| **10.Ohikhuare 2020 [Nigeria]** | Juvenile delinquents in a juvenile detention centre | n=76 (with a n=76 control sample) | Mean - Male 15·0 years and Female 14·0 years  (9 - 18 years) | N/R | VA - less than 6/6 Myopia: -0·25 D Hyperopia: +0·25 D Astigmatism: Cylindrical Error >0·25 D Anisometropia: 0·50 D | VI - less than 6/6 70% in incarcerated youth, 72·4% had ocular morbidity· [NO anomaly n=9 11·8% incarcerated vs n=33 43·4% control)  RE - 38·2% Other pathologies - 34·8% (Conjunctivitis - 32·3%, lens opacity - 0·66%, RD 0·66%, Glaucoma 1·3%)  Non-incarcerated RE - 44·7% vs Incarcerated 34%  By Eyes: uRE - Right eye - n=24 Delinquents vs n=38 control, Left eyes - n=23 delinquents vs n=38 control Person Level - uRE - n=62 any eye 38·7% in delinquents and 61·3% in control Astigmatism 32·26% (8·06% incarcerated vs 24·2% control) Myopia 29% (1·5% incarcerated vs 14·5% control) Hypermetropia 27·4% (16·1% incarcerated vs 11·3% control)  Glaucoma n=2, 2·63% incarcerated vs n=0 control Lens opacity n=1 1·3% incarcerated vs n=0 control Accommodative issues n=0 incarcerated vs n=4 5·3% control Low vision n=1 1·3% incarcerated vs n=1 1·3% control Retinal Detachment n=1 1·3% incarcerated vs n=0 control | N/R |
| **11.Oliván Gozalvo 2002 [Spain]** | Delinquent female adolescents in a juvenile correctional facility | n=35 | Mean 15·0 years (14 - 17 years) | 0·00%  [100% female] | Requiring referral following visual acuity test with optotypes (including any ocular disorder) | 5·71% (n=2) | N/R |
| **12.Robbins 1983 [USA]** | Adjudicated adolescents referred to a psychiatric clinic by the juvenile judge or probation officer | n=50 (n=25, two groups) (in clinic and not in clinic) | Mean 15·9 years in clinic sample and 15·7 years in non-clinic sample  (14 - 18 years) | 100·00% | Below 20/20 vision,  and apparent serious visual pathology or impaired ocular motor performance | 66% less than 20/20 (n=33),  12% (n=6) with serious visual pathology (monocular blindness and scotoma),  18% (n=9) with inadequate oculomotor performance,  44% (n=22) problem with tracking in the left eye, 28% (n=14) had problems with tracking in right eye,  24% (n=12) with severe convergence problems·  Does not report the combined prevalence of VI conditions·  Criminal History - Repeat offender clinic sample n=17 and non-clinic n=10 (Chi square 5·30, p<0·05) | N/R |
| **13.Shandra 2012 [USA]** | Youth at household level (population based, youth with a history of arrest included in the review) | n=7232 | N/R but specified as adolescents around 16 years of age | 50·70% | N/R | Overall prevalence of any sensory impairment (VI or HI) 11%·  Proportion with impairments among the arrested youth: -Blindness - (Total n=21 x 28·57%) -Vision difficulties - (Total n=546 x 9·52%) Overall proportion arrested 12·9% Any sensory impairment (vision or hearing) 11% | N/R |
| **14.Snow 1983 [USA]** | Juvenile Delinquents at a juvenile court centre | n=253 | N/R but specified as juveniles | 77·86% | Based on referral criteria: 1) VA 20/40 or poorer acuity with either eye or a two-line difference between the two eyes 2) Any strabismus and phorias 3) Defective stereo acuity | Any vision problem: Male n=106, Female n=40 Overall visual impairment by 20/30:  Male n=98 (by eyes), Female n=65 (by eyes) Refractive error problems: Male n=37, Female n=25 Ocular muscles imbalances:  Male n=81, Female n=25 Both RE and Ocular muscle imbalances:  Male n=12, Female n=10 Suspected ocular pathology: Male n=0, Female n=1  [*Need to cross check following data with control sample article] Visual acuity: Referral rate: Age 10-14 yrs:  27% delinquents vs 10% non-delinquents· Age 15-19 yrs: 26% delinquent vs 20% non-delinquents Poor ocular muscle coordination: Referral rate:  Age 10-14 yrs:  42% delinquent vs 10% non-delinquents Age 15-19 yrs:  43% delinquent vs 10% non-delinquents | Referral rates due to ocular problems were 4 times higher in the delinquents compared to a control· |
| **15.Weindling 1986 [UK]** | Delinquent adolescents in an intermediate treatment centre | n=48 (n=24 Delinquent vs n=24 non-Delinquent) | Mean - Delinquents 15·3 years and control 15·7 years | 100·00% | N/R | Failed vision test: Delinquent n=9 (38%), Control n=7 (29%) Spectacle wearing:  Delinquents n=1 (lost spectacles) vs Control n=5 | None of the delinquent boys were aware that they had a vision problem  Spectacle usage p<0·01 |
| **16.Wong 1976 [USA]** | Juvenile delinquents in a juvenile detention centre | n=633 | Mean 14·65 years  (7 - 18 years) | 76·90% | Referral after failing at least one of Modified Clinical Technique Test VA - 20/40 or less in either eye Hyperopia +1·5D or more Myopia -0·50D or more Astigmatism +/-1·00D or more Anisometropia +/-1·00D or more Coordination problems at neat and at distance | Rate of referral based on visual impairment 29·5% (n=186, n=62 females and n=124 males) (n=137, 68·8% due to RE) (Other ophthalmological n=28, 14·1%) (Muscle coordination n=13, 6·5%) (Amblyopia n=7, 3·5%) [This is not by person number, but eyes]  RE Distribution: Myopia n=70 (25·5%) Myopia with astigmatism n=96 (35·0%) Hyperopia n=16 (5·6%) Hyperopia with astigmatism n=72 (26·3%) Astigmatism n=18 (6·6%) Emmetropia n=2 (0·7%) | N/R |

**Supplementary File 3 - Table 2. Data extraction table of studies describe hearing loss or ear disease among the incarcerated youth**

| **Author, Year, Country** | **Type of population and setting** | **Sample Size** | **Mean Age and Age Range** | **Proportion of Males** | **Definition of Hearing Impairment** | **Prevalence of Hearing Impairment (HI)** | **Healthcare Access Data** |
| --- | --- | --- | --- | --- | --- | --- | --- |
| **1.Belenchia 1983 [USA]** | Prisoners in a State Penitentiary | n=85 | Mean 20·73 years (16-25) | 70·59% | No response at 20 dB HL at 1, 2, 4, & 6 kHz  HI cut off 20 dB HL | Overall prevalence of HI 48·5% for the total 136 participants including ages from 16-60 years  [*Data for the 16-20 age group and the 21-25 age group was given and sample size adjusted in the meta-analysis] | N/R |
| **2.Cozad 1966 [USA]** | Industrial schools (for institutionalized children) | n=300 | Mean 15·0 years for boys and 16·0 years for girls  (10-18) | 70·60% | No response at 10dB for 1, 2, or 6k· No response at 20dB for 4k  HI cut-off: 10dB for 1, 2, 6k; 20dB for 4k | 24·33 (n=73) referred on the hearing screening  Comprehensive audiometry testing (air and bone) of participants failing the screening was completed and showed 72·6% (n=53) of those screened had sensorineural hearing loss  27·4% (n=20) had mixed hearing loss, and no individual (n=0) had conductive hearing loss | N/R |
| **3.He 2019 [Australia]** | Aboriginal children around the age of 10 as that is the minimum legal age of criminal responsibility in the Northern Territory | n=1533 | N/R | 49·20% | First reported audiogram in the data set with a 3-frequency PTA (0·5, 1· & 2 kHz) in both ears Only conductive and mixed hearing losses were included | Overall prevalence of HI 48·08% (n=737)  21·14% (n=324) had unilateral hearing loss (UHL)  19·63% (n=301) had mild hearing impairment  7·31% (n=112) had moderate or worse hearing impairment  Among children with a record of being found guilty of an offence (n=191), 55·6% of boys and 36·7% of girls had UHL/hearing loss  For boys, those with moderate (or worse) hearing loss had the highest cumulative proportion of youth offending (40·0%; 95% CI: 24·8–60·1%), followed by boys with mild (28·9%; 95% CI: 20·9–39·1), UHL (28·7%; 95% CI: 20·3–39·6), and normal hearing (23·2%; 95% CI:18·2–29·2)· For girls, those with normal hearing had the highest cumulative proportion of youth offending (10·1%; 95% CI: 7·1–14·2), followed by girls with mild (7·4%; 95% CI: 3·7–4·7), moderate (or worse) (7·2%; 95% CI:2·3–21·1) and UHL (5·9%; 95% CI:2·7– 12·2)  The risk of offending was significantly higher in boys with a record of moderate or worse HI (HR: 1·77, 95% CI:1·05–2·98, p = 0·031) and those with mild HI (HR: 1·54, 95% CI:1·06–2·23, p = 0·023) than in boys with normal hearing in the univariate analysis  These associations were attenuated when child maltreatment, school factors (school attendance and enrolment by mother), and community fixed-effects were added to the multivariable model, with no evidence for an association between HI and offending (moderate HI, HR: 1·43, 95% CI = 0·78–2·62, p = 0·252; mild HI, HR: 1·37, 95% CI = 0·83–2·26, p = 0·215) | N/R |
| **4.Holmes 1996 [USA]** | Incarcerated juveniles in a detention centre | n=226 | Mean 15·3 years  (9-18) | 76·50% | Either otoscopy (inability to view tympanum), tympanometry (<= -200mm H2O or noncompliant middle ear system), or no response at 25dB HL to pure-tone stimuli at any frequency (1K, 2K, 4K, and 6K in either ear)  HI cut-off: 25dB HL for 1K, 2K, 4K, and 6K | 26·11% (n=59) failed pure tone screening  35·4% (n=80) failed one, two, or all of tests (otoscopy, tympanometry, pure tone screening)  9·7% (n=22) failed otoscopy  7·5% (n=17) failed tympanometry | N/R |
| **5.Indig 2011**  **[Australia]**  **(NSW Department of Juvenile Justice 2009 Report)** | Juvenile delinquents in 10 juvenile detention centers throughout Australia | n=278 | Mean 17·0 years | 88·0% | Not reported on cut offs for what is considered normal | 18·35% (n=51) had hearing loss  32% (n=89) had unilateral hearing loss  44·24% (n=123) had abnormal ear canals in at least one ear during otoscopy· Abnormalities included red ear canals (n=23), narrow ear canals (n=12), discharge (n=18), wax occluding (n=58), and other (n=12)  14·03% (n=39) had abnormal eardrums during otoscopy in at least one ear· Abnormalities included wet perforation (n=3), dry perforation (n=11), tympanosclerosis/scarring (n=14), acute otitis media (n=1), Otitis media with effusion (n=4), and other (n=6)  18·12% (n=52) had eardrums that could not be visualized due to wax impaction, discharge, or swollen ear canals· | N/R |
| **6.Lount 2017 [New Zealand]** | Male juvenile delinquents in a detention center | n=33 | Mean 16·04 years  (14-17) | 100·0% | PTA at 500, 1000, 2000, and 4000 Hz was greater than 15 dB HL  HI cut-off: 15 dB HL  Self-report of hearing difficulty: *“always easy”, “sometimes difficult”, & “always difficult”*  Type A tympanogram considered normal middle ear function· | For pure tone audiometry, 24% (n=8) of youth offenders vs· 5% (n=2) of controls had slight hearing loss based on their PTA· 6% (n=2) of youth offenders vs· no controls had mild hearing loss based on their PTA  For tympanometry, 48% (n=16) of youth offenders vs· 62% (n=24) of controls had an abnormal tympanogram (other than Type A) in at least one ear·  For self-report of hearing, 36% (n=12) of youth offenders vs· 44% (n=17) of the control group reported hearing as “sometimes difficult”. 3% (n=1) of youth offenders reported hearing as “always difficult” | N/R |
| **7.Morgan 1979 [USA]** | Juvenile detention centers throughout the US | n=26,740 | N/R | 86·0% | N/R | Any impairment 42·4% (National average 12·3%) (Male n=9084, Female n=1431) (n=11,333/26,740)  Hearing handicap (HH) n= 365 (1·36% in incarcerated youth compared to 0·5% in controls· | N/R |
| **8.Omokanye 2022 [Nigeria]** | Young adults in a juvenile correctional facility | n=135 | Mean age: 19·0 years [Range 15-21 years] | N/R | Cut off for normal hearing threshold ≤25dB | Prevalence of hearing loss (PTA, air conduction): 19·2% (n=26) in better and 34·8% (n=48) in worse ear inmates vs 0·0% (n=0) in better and 2·2% (n=13) in worse ear of controls  Conductive hearing loss 24·4% (n=33) inmates vs 9·4% (n=13) controls; Sensory-neural hearing loss 2·2% (n=3) and mixed hearing loss 9·0% (n=12) among inmates and 0% in controls  Self-reported hard of hearing: 31·9% (n=43) inmates vs 8·7% (n=12) controls  Otoscopic Findings: Impacted wax (cerumen) 13·3% (n=18) inmates vs 7·4% (n=10) control; perforated or neo-membrane 8·0% (n=11) inmates vs 0·7% (n=1) controls; Dull tympanic membrane light reflex 26·0% (n=35) inmates vs 14·0% (n=19) controls; Retracted position of tympanic membrane 12·6% (n=17) inmates vs 0·7% (n=1) controls | N/R |
| **9.Shandra 2012 [USA]** | Adolescents in the National Longitudinal Survey of Youth 1997  (Youth with a history of arrest included in the review) | n=933 | N/R | 50·70% | Self-report of hearing difficulty  No objective measure of hearing was completed· | Overall prevalence of any sensory impairment (VI or HI) 11%·  Proportion with impairments among the arrested youth:  Hearing difficulty - (Total n=85, 16·47%)  Overall proportion arrested 12·9%  Any sensory impairment (vision or hearing) 11% | N/R |
| **10.Wagner 1983 [USA]** | Prisoners in Purdy Treatment Center for Women | n=50  [n=18 <24 years] | Mean 27·64 years  (18-44) | 0·00% | Failure to hear at screening level for any one frequency  20 dB HL at 1 kHz, 2 kHz and 25 dB HL at 4 kHz  HI cut-off: 20 dB HL at 1 & 2 kHz and 25 dB HL at 4 kHz | 16·0% (n=8) referred on the hearing screening  [Data stratified by age range 18-20 years and 21-23 years] | N/R |
| **11.Weindling 1986 [UK]** | Juvenile delinquents in a Juvenile detention center | n=24 | Mean 15·3 years | 100% | Failed to hear at >= 30 dB on at least two frequencies from 500-4000 Hz | 33·0% (n=8) referred on the hearing screening compared to 16% (n=4) of the control group | N/R |

**Supplementary File 4 - Table 1. Study quality appraisal and risk of bias assessment domains* of studies included in the systematic review**

| **Author, Year** | ***1) Were the criteria for inclusion in the sample clearly defined?*** | ***2) Were the study subjects and the setting described in detail?*** | ***3) Was the exposure measured in a valid and reliable way?*** | ***4) Were objective, standard criteria used for measurement of the condition?*** | ***5) Were confounding factors identified?*** | ***6) Were strategies to deal with confounding factors stated?*** | ***7) Were the outcomes measured in a valid and reliable way?*** | ***8) Was appropriate statistical analysis used?*** |
| --- | --- | --- | --- | --- | --- | --- | --- | --- |
| **1.Barnes 1978** | No | No | Yes | Yes | Yes | No | Yes | Unclear |
| **2.Belenchia 1983** | Yes | Yes | Yes | Yes | No | NA | NA | NA |
| **3.Brian 2011** | Yes | Yes | Yes | Yes | NA | NA | Yes | Yes |
| **4.Cozad 1966** | Yes | Yes | Yes | Yes | No | NA | NA | NA |
| **5.Dzik 1966** | Yes | Yes | No | Unclear | NA | NA | Yes | Unclear |
| **6.Harrie 2016** | Yes | Yes | Yes | Yes | Yes | No | Yes | Yes |
| **7.Harris 1989** | Yes | Yes | Yes | Yes | No | No | Yes | Yes |
| **8.He 2019** | Yes | Yes | Yes | Yes | Yes | Yes | Yes | Yes |
| **9.Holmes 1996** | Yes | Yes | Yes | Yes | No | NA | NA | NA |
| **10.Johnson and Zaba 1999** | Yes | Yes | Yes | Yes | No | NA | Yes | Yes |
| **11.Kaseno 1985** | No | Yes | No | No | NA | NA | No | Unclear |
| **12.Lount 2017** | Yes | Yes | Yes | Yes | No | NA | Yes | Yes |
| **13.Morgan 1979** | No | No | No | No | No | NA | NA | NA |
| **14.NSW Report 2009** | Yes | Yes | Yes | Yes | NA | NA | Yes | Unclear |
| **15.Olivan-Gozalvo 2002** | Unclear | Yes | Yes | Yes | NA | NA | Yes | Yes |
| **16.Ohikhuare 2020** | Yes | Yes | Yes | Yes | No | No | Yes | Yes |
| **17.Omokanye 2022** | Yes | Yes | Yes | Yes | No | NA | Yes | Yes |
| **18.Robbins 1983** | Yes | Yes | Yes | Yes | NA | NA | Yes | Yes |
| **19.Shandra 2012** | Yes | Yes | Yes | Yes | Yes | Yes | Yes | Yes |
| **20.Snow 1983** | Unclear | Yes | Yes | Yes | No | No | Yes | Unclear |
| **21.Wagner 1983** | Yes | Yes | Yes | Yes | No | NA | NA | NA |
| **22.Weilding 1986** | No | No | Unclear | Unclear | No | No | Unclear | Unclear |
| **23.Wong 1976** | Yes | Yes | Yes | Yes | No | No | Yes | Yes |

(*Tool - Joanna Brigs Institute critical appraisal tools)

**Supplementary File 5 - Table 1. Summary of vision impairment/eye disease and hearing impairment/ear disease prevalence data extracted for narrative synthesis and meta-analysis**

| **Author, Year** | **Country** | **Sample Size** | **Control Sample** | **Mean Age**  **(Years)** | **Proportion of Male** | **Type of Defect** | **Defect Variable** | **Main Var:** | **Prisoners Cases**  **(n=)** | **Prisoners Normal (n=)** | **Prisoners Prevalence (%)** | **Control Cases**  **(n=)** | **Control Normal (n=)** | **Control Prevalence (%)** |
| --- | --- | --- | --- | --- | --- | --- | --- | --- | --- | --- | --- | --- | --- | --- |
| **Belenchia 1983** | USA | 85 | · | 20·8 | 70·6% | Hearing Impairment | HI | Hearing | 41 | 44 | 48·5 | · | · | · |
| **Cozad 1966** | USA | 300 | · | · | 70·6% | Hearing Impairment | HI | Hearing | 73 | 227 | 24·3 | · | · | · |
| **He 2019** | Australia | 1533 | · | 10·2 | 49·2% | Hearing Impairment (Unilateral) | HI | Hearing | 324 | 1209 | 21·1 | · | · | · |
| **He 2019** | Australia | 1533 | · | 10·2 | 49·2% | Hearing Impairment (Mild) | HI | Hearing | 301 | 1232 | 19·6 | · | · | · |
| **He 2019** | Australia | 1533 | · | 10·2 | 49·2% | Hearing Impairment (Moderate-Worse) | HI | Hearing | 112 | 1421 | 7·30 | · | · | · |
| **Holmes 1996** | USA | 226 | · | 15·3 | 76·5% | Hearing Impairment | HI | Hearing | 59 | 167 | 26·1 | · | · | · |
| **Holmes 1996** | USA | 226 | · | 15·3 | 76·5% | Middle Ear Dysfunction | HI-Other | Hearing | 17 | 209 | 7·52 | · | · | · |
| **Holmes 1996** | USA | 226 | · | 15·3 | 76·5% | Occluded Ear Canal | HI-Other | Hearing | 22 | 204 | 9·73 | · | · | · |
| **Lount 2017** | New Zealand | 33 | 39 | 16·0 | 100·0% | Hearing Impairment | HI | Hearing | 10 | 23 | 30·3 | 2 | 37 | 5·41 |
| **Morgan 1979** | USA | 26740 | 26740 | · | 86·0% | Hearing Impairment | HI | Hearing | 364 | 26376 | 1·36 | 134 | 26606 | 0·50 |
| **Morgan 1979** | USA | 26740 | 26740 | · | 86·0% | Deafness | DF | Hearing | 8 | 26732 | 0·03 | 19 | 26721 | 0·07 |
| **NSW Report 2009** | Australia | 278 | · | 17 | 88·4% | Hearing Impairment (Any degree) | HI | Hearing | 140 | 138 | 50·4 | · | · | · |
| **NSW Report 2009** | Australia | 287 | · | 17 | 88·4% | Abnormal Ear Drums | HI-Other | Hearing | 20 | 267 | 6·95 | · | · | · |
| **Omokanye 2022** | Nigeria | 135 | 135 | 19 | · | Hearing Impairment (Better ear) | HI | Hearing | 26 | 109 | 19·2 | 0 | 135 | 0 |
| **Shandra 2012** | USA | 933 | · | · | 50·7% | Hearing Impairment | HI | Hearing | 14 | 919 | 16·5 | · | · | · |
| **Wagner 1983** | USA | 18 | · | · | 0·00% | Hearing Impairment | HI | Hearing | 3 | 15 | 16·0 | · | · | · |
| **Weindling, 1986** | UK | 24 | · | 15·3 | 100·0% | Hearing Impairment | HI | Hearing | 8 | 16 | 33·0 | 4 | 20 | 17·0 |
| **Barnes 1978** | Ireland | 100 | 115 | · | 100·0% | Near Vision Impairment | NVI/RE | Vision | 2 | 98 | 2·0 | 0 | 115 | 0 |
| **Barnes 1978** | Ireland | 100 | 115 | · | 100·0% | Distant Vision Impairment | DVI | Vision | 3 | 97 | 3·0 | 1 | 114 | 1·0 |
| **Barnes 1978** | Ireland | 100 | 115 | · | 100·0% | Enucleated Eye | VI-Other | Vision | 2 | 98 | 2·0 | 0 | 115 | 0 |
| **Dzik 1966** | USA | 125 | · | · | 100·0% | Distant Vision Impairment | DVI | Vision | 63 | 62 | 50·4 | · | · | · |
| **Harrie 2016** | USA | 1632 | 1792 | · | 69·8% | Uncorrected Refractive Errors | NVI/RE | Vision | 569 | 1063 | 34·9 | 394 | 1393 | 21·9 |
| **Harrie 2016** | USA | 1632 | · | · | 69·8% | Amblyopia | VI-Other | Vision | 22 | 1610 | 1·35 | · | · | · |
| **Harries 1989** | USA | 132 | · | 16·7 | 90·0% | Distant Vision Impairment | DVI | Vision | 8 | 124 | 6·45 | · | · | · |
| **Johnson and Zaba 1999** | USA | 50 | 44 | 19 | 98·0% | Near Vision Impairment | NVI/RE | Vision | 8 | 42 | 16·0 | 0 | 44 | 0 |
| **Johnson and Zaba 1999** | USA | 50 | 44 | 19 | 98·0% | Distant Vision Impairment | DVI | Vision | 2 | 48 | 4·0 | 6 | 38 | 14·0 |
| **Kaseno 1985** | USA | 1000 |  | 16·2 | 90·0% | Uncorrected Refractive Errors | NVI/RE | Vision | 90 | 910 | 9·02 | · | · | · |
| **Morgan 1979** | USA | 26740 | 26740 | · | 86·0% | Vision Problems | VI-Other | Vision | 425 | 26315 | 1·59 | 27 | 26713 | 0·10 |
| **Morgan 1979** | USA | 26740 | 26740 | · | 86·0% | Blindness | BL | Vision | 0 | 26740 | 0 | 16 | 26724 | 0·06 |
| **NSW Report 2009** | Australia | 361 | · | 17 | 88·4% | Vision Problems | VI-Other | Vision | 24 | 337 | 6·65 | · | · | · |
| **Ohikhuare 2020** | Nigeria | 76 | 76 | 15 | · | Uncorrected Refractive Errors | NVI/RE | Vision | 26 | 50 | 34·0 | 34 | 42 | 44·7 |
| **Ohikhuare 2020** | Nigeria | 76 | 76 | 15 | · | Vision Problems | VI-Other | Vision | 5 | 71 | 6·58 | 5 | 71 | 6·58 |
| **Olivan-Gozalvo 2002** | Spain | 35 | · | 15 | 0·00% | Vision Problems | VI-Other | Vision | 2 | 33 | 5·71 | · | · | · |
| **Robbins 1983** | USA | 50 | · | 15·8 | 100·0% | Vision Problems | VI-Other | Vision | 33 | 17 | 66·0 | · | · | · |
| **Robbins 1983** | USA | 50 | · | 15·8 | 100·0% | Monocular Blindness | BL | Vision | 6 | 44 | 12·0 | · | · | · |
| **Shandra 2012** | USA | 933 | · | · | 50·7% | Vision Problems | VI-Other | Vision | 52 | 881 | 5·57 | · | · | · |
| **Shandra 2012** | USA | 933 | · | · | 50·7% | Blindness | BL | Vision | 6 | 927 | 0·64 | · | · | · |
| **Snow 1983** | USA | 253 | 253 | · | 77·8% | Uncorrected Refractive Errors | NVI/RE | Vision | 62 | 191 | 24·5 | 37 | 216 | 14·8 |
| **Weindling 1986** | UK | 24 | 24 | 15·3 | 100·0% | Vision Problems | VI-Other | Vision | 9 | 15 | 37·5 | 7 | 17 | 29·2 |
| **Wong 1976** | USA | 633 | 633 | 14·6 | 76·9% | Vision Problems | VI-Other | Vision | 186 | 447 | 29·4 | 176 | 457 | 27·8 |
| **Brian G 2011^‡^** | PapuaNewGuinea | 49 | · | 21 | 95·9% | Near Vision Impairment | NVI/RE | Vision | 4 | 45 | 8·1 | · | · | · |
| **Brian G 2011^‡^** | PapuaNewGuinea | 49 | · | 21 | 95·9% | Distant Vision Impairment | DVI | Vision | 3 | 46 | 6·1 | · | · | · |

[‡ Brian 2011 - Author provided age stratified data included in the review]

**Supplementary File 6 - Forest plot of odds of prevalence of vision/hearing impairment among the incarcerated youth compared to a non-offended control sample from population and leave-one-out meta-analysis of vision impairment**

**
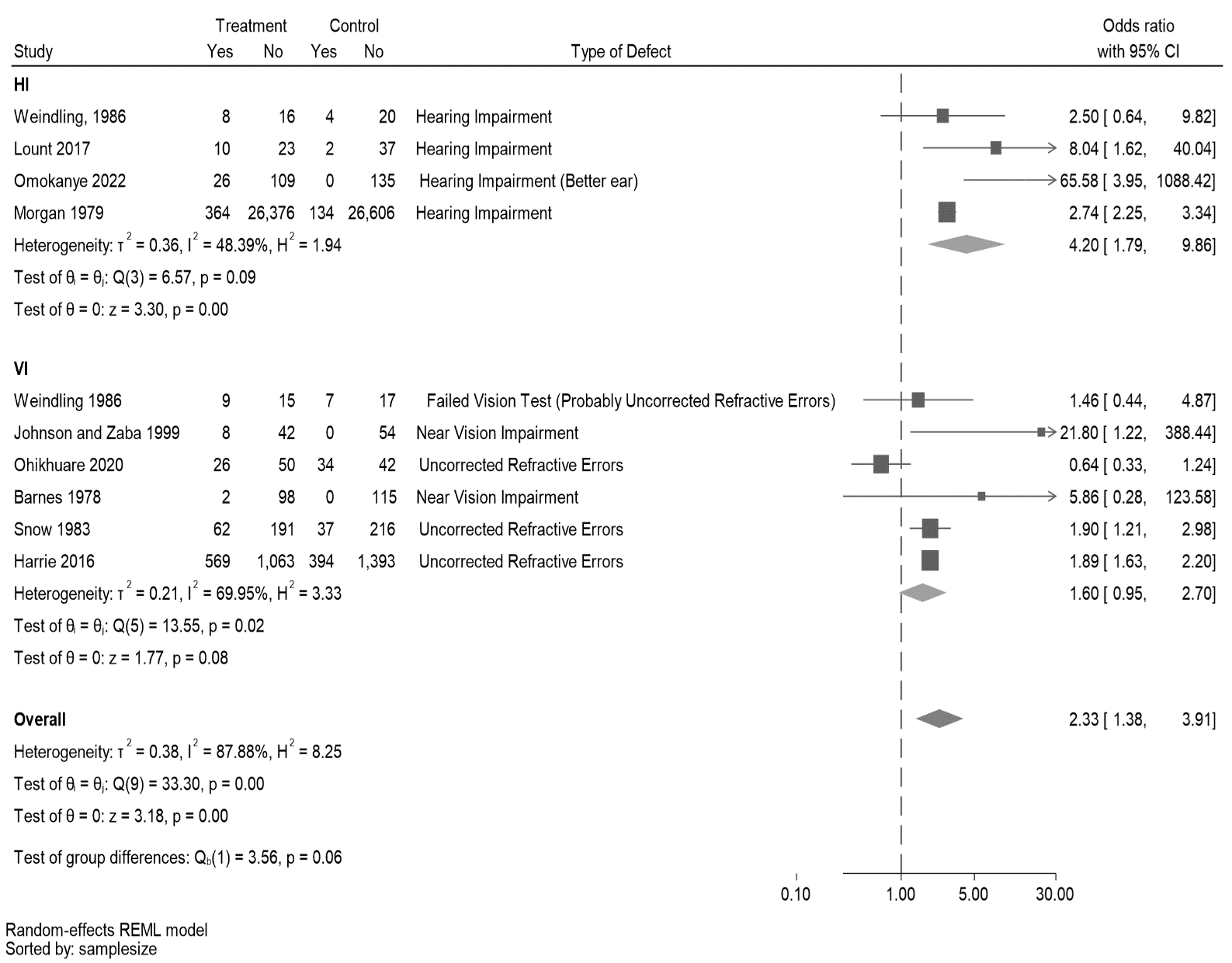
**

**Supplementary File 6-Figure 1.** Forest plot of odds of prevalence of vision/hearing impairment among the incarcerated youth compared to a non-offended control sample from population (with out the leave-one-out analysis)

**Supplementary File 6-Figure 2.** Forest plot of odds of prevalence of vision impairment among the incarcerated youth compared to a non-offended control sample from population using leave-one-out analysis*

**(odds ratio in Figure 2 provides the final summary estimate for vision impairment when leave-out each study; the central line represents the final summary estimate of OR=1.60 if not conducted the leave-one-out analysis)*
